# Quantifying rates of HIV-1 flow between risk groups and geographic locations in Kenya: A country-wide phylogenetic study

**DOI:** 10.1093/ve/veac016

**Published:** 2022-03-03

**Authors:** George M Nduva, Frederick Otieno, Joshua Kimani, Elizabeth Wahome, Lyle R McKinnon, Francois Cholette, Maxwell Majiwa, Moses Masika, Gaudensia Mutua, Omu Anzala, Susan M Graham, Larry Gelmon, Matt A Price, Adrian D Smith, Robert C Bailey, Guy Baele, Philippe Lemey, Amin S Hassan, Eduard J Sanders, Joakim Esbjörnsson

**Affiliations:** Department of Translational Medicine, Lund University, Faculty of Medicine, Lund University, Box 117 SE-221 00 Lund, Sweden; Kenya Medical Research Institute-Wellcome Trust Research Programme, KEMRI-Center For Geographic Medicine Research, P.O. Box 230-80108, Kilifi, Kenya; Nyanza Reproductive Health Society, United Mall, P.O. Box 1764, Kisumu, Kenya; Department of Medical Microbiology, University of Nairobi, P.O. Box 30197-00100, Nairobi, Kenya; Department of Medical Microbiology and Infectious Diseases, University of Manitoba, Max Rady College of Medicine, Room 543-745 Bannatyne Avenue, University of Manitoba (Bannatyne campus), Winnipeg MB R3E 0J9, Canada; Kenya Medical Research Institute-Wellcome Trust Research Programme, KEMRI-Center For Geographic Medicine Research, P.O. Box 230-80108, Kilifi, Kenya; Department of Medical Microbiology, University of Nairobi, P.O. Box 30197-00100, Nairobi, Kenya; Department of Medical Microbiology and Infectious Diseases, University of Manitoba, Max Rady College of Medicine, Room 543-745 Bannatyne Avenue, University of Manitoba (Bannatyne campus), Winnipeg MB R3E 0J9, Canada; Centre for the AIDS Programme of Research in South Africa (CAPRISA), Doris Duke Medical Research Institute, Nelson R Mandela School of Medicine, University of KwaZulu-Natal, Private Bag X7, Congella 4013, South Africa; Department of Medical Microbiology and Infectious Diseases, University of Manitoba, Max Rady College of Medicine, Room 543-745 Bannatyne Avenue, University of Manitoba (Bannatyne campus), Winnipeg MB R3E 0J9, Canada; National Microbiology Laboratory at the JC Wilt Infectious Diseases Research Centre, Public Health Agency of Canada, 745 Logan Avenue, Winnipeg, Canada; Kenya Medical Research Institute/Center for Global Health Research, KEMRI-CGHR, P.O. Box 20778-00202, Kisumu, Kenya; Faculty of Health Sciences 3RD Floor Wing B, KAVI Institute of Clinical Research, University of Nairobi, P.O. Box 19676-00202, Nairobi, Kenya; Faculty of Health Sciences 3RD Floor Wing B, KAVI Institute of Clinical Research, University of Nairobi, P.O. Box 19676-00202, Nairobi, Kenya; Faculty of Health Sciences 3RD Floor Wing B, KAVI Institute of Clinical Research, University of Nairobi, P.O. Box 19676-00202, Nairobi, Kenya; Kenya Medical Research Institute-Wellcome Trust Research Programme, KEMRI-Center For Geographic Medicine Research, P.O. Box 230-80108, Kilifi, Kenya; Department of Epidemiology, University of Washington, Office of the Chair, UW Box # 351619, Seattle, DC, USA; Department of Medical Microbiology, University of Nairobi, P.O. Box 30197-00100, Nairobi, Kenya; Department of Medical Microbiology and Infectious Diseases, University of Manitoba, Max Rady College of Medicine, Room 543-745 Bannatyne Avenue, University of Manitoba (Bannatyne campus), Winnipeg MB R3E 0J9, Canada; IAVI Global Headquarters, 125 Broad Street, 9th Floor, New York, NY 10004, USA; Department of Epidemiology and Biostatistics, University of California, Mission Hall: Global Health & Clinical Sciences Building, 550 16th Street, 2nd Floor, San Francisco, CA 94158-2549, USA; Nuffield Department of Medicine, The University of Oxford, Old Road Campus, Headington, Oxford OX3 7BN, UK; Nyanza Reproductive Health Society, United Mall, P.O. Box 1764, Kisumu, Kenya; Division of Epidemiology and Biostatistics, University of Illinois at Chicago, 1603 W Taylor St, Chicago, IL 60612, USA; KU Leuven Department of Microbiology, Immunology and Transplantation, Rega Institute, Laboratory of Clinical and Evolutionary and Computational Virology, Rega-Herestraat 49-box 1040, Leuven 3000, Belgium; KU Leuven Department of Microbiology, Immunology and Transplantation, Rega Institute, Laboratory of Clinical and Evolutionary and Computational Virology, Rega-Herestraat 49-box 1040, Leuven 3000, Belgium; Department of Translational Medicine, Lund University, Faculty of Medicine, Lund University, Box 117 SE-221 00 Lund, Sweden; Kenya Medical Research Institute-Wellcome Trust Research Programme, KEMRI-Center For Geographic Medicine Research, P.O. Box 230-80108, Kilifi, Kenya; Kenya Medical Research Institute-Wellcome Trust Research Programme, KEMRI-Center For Geographic Medicine Research, P.O. Box 230-80108, Kilifi, Kenya; Nuffield Department of Medicine, The University of Oxford, Old Road Campus, Headington, Oxford OX3 7BN, UK; Department of Translational Medicine, Lund University, Faculty of Medicine, Lund University, Box 117 SE-221 00 Lund, Sweden; Nuffield Department of Medicine, The University of Oxford, Old Road Campus, Headington, Oxford OX3 7BN, UK

**Keywords:** HIV-1, key populations, molecular epidemiology, transmission

## Abstract

In Kenya, HIV-1 key populations including men having sex with men (MSM), people who inject drugs (PWID) and female sex workers (FSW) are thought to significantly contribute to HIV-1 transmission in the wider, mostly heterosexual (HET) HIV-1 transmission network. However, clear data on HIV-1 transmission dynamics within and between these groups are limited. We aimed to empirically quantify rates of HIV-1 flow between key populations and the HET population, as well as between different geographic regions to determine HIV-1 ‘hotspots’ and their contribution to HIV-1 transmission in Kenya. We used maximum-likelihood phylogenetic and Bayesian inference to analyse 4058 HIV-1 *pol* sequences (representing 0.3 per cent of the epidemic in Kenya) sampled 1986–2019 from individuals of different risk groups and regions in Kenya. We found 89 per cent within-risk group transmission and 11 per cent mixing between risk groups, cyclic HIV-1 exchange between adjoining geographic provinces and strong evidence of HIV-1 dissemination from (i) West-to-East (i.e. higher-to-lower HIV-1 prevalence regions), and (ii) heterosexual-to-key populations. Low HIV-1 prevalence regions and key populations are sinks rather than major sources of HIV-1 transmission in Kenya. Targeting key populations in Kenya needs to occur concurrently with strengthening interventions in the general epidemic.

## 1. Introduction

The world is off-track on the United Nations Programme on HIV and AIDS (UNAIDS) objective to reduce the global HIV-1 incidence rate, with an estimated 1.7 million new HIV-1 infections in 2019 (Joint United Nations Programme on HIV/AIDS (UNAIDS) 2020). To fast-track reduction in global HIV-1 incidence whilst also achieving efficiency gains, UNAIDS directs national governments to invest strategically in HIV-1 programmes. This includes directing treatment and prevention to HIV-1 key populations (defined as UNAIDS as gay men and other men who have sex with men [MSM], people who inject drugs [PWID], sex workers [FSW], transgender people, and sex partners of key populations) (Kelly et al. 2018). An approach to inform decision-making is to identify populations populations that contribute with a disproportionate number of infections in local epidemic and to eliminate structural and social barriers to health service delivery among key populations (Smith et al. 2009; Anderson et al. 2014).

In North America and European settings, the HIV-1 epidemic mainly affects HIV-1 key populations, and the availability of large numbers of HIV-1 genetic sequences and associated patient risk group information have allowed extensive characterisation of HIV-1 networks (Esbjörnsson et al. 2016; Poon et al. 2016; Ratmann et al. 2016). In contrast, in sub-Saharan Africa (accounting for 65 per cent of all new HIV-1 infections globally), the HIV-1 epidemic mainly affects the heterosexual population (HET). However, pockets of concentrated sub-epidemics involving high-risk groups have also been described (Joint United Nations Programme on HIV/AIDS (UNAIDS) 2018; Abeler-Dörner et al. 2019; Nduva et al. 2021). Additionally, there is evidence of overlapping sexual networks and phylogenetic linkages between HIV-1 key populations and HET (Nduva et al. 2021). However, the scarcity of HIV-1 sequences from key populations has limited phylogenetic assessment of HIV-1 transmsission within and between key populations and lower-risk populations in sub-Saharan Africa.

Kenya has the fifth-largest number of people with HIV-1 in the world, and the early HIV-1 epidemic in the country was defined exclusively as heterosexual and involving FSW and long-distance truck drivers (Kreiss et al. 1986; National AIDS and STI Control Programme (NASCOP) 2020). As a consequence, governmental HIV-1 surveillance did not focus on other marginalised key populations such as MSM and PWID (Sanders et al. 2007; Smith et al. 2009; Makofane et al. 2020). The Kenyan Ministry of Health has reported high HIV-1 prevalence among key populations (29.3 per cent among FSW, 18.2 per cent among MSM and 18.2 per cent among PWID, compared to 4.5 per cent in the general epidemic) (Kenya National AIDS Control Council 2009; National AIDS and STI Control Programme 2017). As a consequence, directed programmes for key populations have been initiated based on the assumption that they contribute with a disproportionate number of infections to the larger HIV-1 transmission network in the nationwide epidemic (National AIDS and STI Control Programme 2017; Kenya National AIDS Control Council 2019). However, phylogenetic studies in Coastal Kenya have suggested that most HIV-1 transmissions occur within risk groups (with only 15 per cent of the identified clusters reflecting mixing between MSM, FSW, and HET in Coastal Kenya) (Bezemer et al. 2014; Nduva et al. 2020). Moreover, to the best of our knowledge, no study has empirically assessed the rates of HIV-1 flow between key populations and the heterosexual population in Kenya. Also, spatial mapping of the Kenyan epidemic has revealed extensive geographic heterogeneity with HIV-1 prevalence ranging from less than 1 per cent in the North Eastern province to more than 20 per cent around the shores of Lake Victoria in the Western regions of the country (National AIDS and STI Control Programme (NASCOP) 2020). Such spatial differences in HIV-1 distribution likely influence HIV-1 diffusion dynamics (Faria et al. 2014; Grabowski et al. 2020), but HIV-1 transmission rates between different geographic areas in Kenya are still unknown.

Phylodynamic analysis has been widely used to determine HIV-1 networks, reconstruct virus historical spatial dissemination, as well as assessing rates of virus flow between populations with varying HIV-1 prevalence (Esbjörnsson et al. 2011, 2016; Bezemer et al. 2014; De Oliveira et al. 2017; Sallam et al. 2017; Hassan et al. 2018; Bbosa et al. 2019; Faria et al. 2019; Nazziwa et al. 2020; Nduva et al. 2020; Ratmann et al. 2020). However, due to the scarcity of HIV-1 sequences from key populations, phylogeographic assessment of HIV-1 transmission rates between populations are rare in sub-Saharan Africa (Bbosa et al. 2019). Here, we combined HIV-1 phylogenetic and epidemiological data to reconstruct HIV-1 networks and to empirically quantify rates of HIV-1 flow between risk groups and geographic regions to identify and determine the contribution of HIV-1 ‘hotspots’ in sustaining HIV-1 transmission in Kenya. We hypothesised that virus flow would be predominantly from high prevalence ‘hotspots’ to the lower prevalence populations.

## 2. Methods

### 2.1 Study population and sequence dataset

New HIV-1 *pol* sequences were generated from blood plasma obtained through studies conducted through the MSM Health Research Consortium—a multi-site collaboration between researchers affiliated with KEMRI-Wellcome Trust (KWTRP) in Coastal Kenya, Nyanza Reproductive Health Society (NRHS) in Western Kenya, Kenya AIDS Vaccine Initiative’s Institute of Clinical Research (KAVI-ICR), and Sex Workers Outreach Program (SWOP) clinics in Nairobi. These included samples from Coast derived from participants in a prospective observational cohort (2006–2019) (Sanders et al. 2013), samples from Nairobi from a respondent-driven sample survey (Smith et al. 2021), and samples from Nyanza derived from the Anza Mapema cohort (2015–2017) (Kunzweiler et al. 2018). Additional nationwide HIV-1 *pol* sequences (2008–2018) were obtained from the national HIV-1 reference laboratory at the Kenya Medical Research Institute (KEMRI)—Centre for Global Health Research.

In addition, all published Kenyan HIV-1 *pol* sequences (1986–2019, corresponding to HXB2 positions 2000–3600) available in the Los Alamos HIV-1 sequence database were retrieved 19 March 2020 (Los Alamos National Laboratory 2019). In cases where more than one sequence per individual was available, the oldest sequence was retained. Newly generated and publicly available sequences were annotated with sampling date, sampling location (province), treatment status, age, sex, and risk group (MSM [men who reported having sex with men]; PWID [men and women who inject drugs]; FSW [female sex workers]; and HET [presumed heterosexuals including men and women for whom risk assessment was not available]). Missing information for published sequences was retrieved from relevant studies or obtained through communication with study authors (Yang et al. 2004; Tovanabutra et al. 2010; Hamers et al. 2011; Hué et al. 2012; Sigaloff et al. 2012; Hassan et al. 2013, 2018; Bezemer et al. 2014; Zeh et al. 2016; Gounder et al. 2017; Onywera et al. 2017).

### 2.2 RNA extraction, DNA amplification, and partial HIV-1 *pol* sequencing

HIV-1 RNA was extracted from blood plasma samples using the RNeasy Lipid Tissue Mini Kit (QIAGEN) with modifications from the manufacturer’s standard protocol (Esbjörnsson et al. 2010). Briefly, 100 µl patient blood plasma was lysed in 1000 μl Qiazol Reagent. Reverse transcription and amplification of partial HIV-1 *pol* gene were performed using the One-Step Superscript III RT/Platinum Taq High Fidelity Enzyme Mix (ThermoFisher Scientific^TM^) with the *pol*-specific primer pair JA269 and JA272 (Hedskog et al. 2010). First-round PCR products were amplified in a nested PCR with DreamTaq Green DNA Polymerase (ThermoFisher Scientific^TM^) using *pol-*specific primers JA271 and JA270 (Hedskog et al. 2010). PCR products were sequenced in both directions with the nested PCR primers using the BigDye terminator kit v1.1 (Applied Biosystems). New HIV-1 *pol* sequences (approximately 1020 nucleotides [nt], HXB2 [K03455] positions 2267–3287) were determined on an ABI PRISM 3130 × 1 Genetic Analyzer (Applied Biosystems).

### 2.3 Population estimates and sampling density

Sampling density (the proportion of genotyped HIV-1 sequences in the estimated number of HIV-infected individuals per geographic location and risk group) was computed based on national HIV-1 prevalence estimates (National AIDS and STI Control Programme 2017; Kenya National AIDS Control Council (NACC) 2018; Kenya National AIDS Control Council 2019; Kenya National Bureau of Statistics 2019); National AIDS and STI Control Programme (NASCOP) 2019;National AIDS and STI Control Programme (NASCOP) 2020).

### 2.4 Subtype analysis

All Kenyan HIV-1 *pol* sequences were combined and aligned with the Los Alamos HIV-1 Group M (subtypes A-K + Recombinants) subtype reference dataset (http://www.hiv.lanl.gov) using the MAFFT algorithm in Geneious Prime 2019 (Larkin et al. 2007). The HIV-1 subtype/circulating recombinant form (CRF) for each sequence was determined by maximum-likelihood (ML) phylogenetic analysis in PhyML using the general time-reversible substitution model with a gamma-distributed rate variation and proportion of invariant sites (GTR + Γ4 + Ι) (Guindon et al. 2010). Branch support was determined by the approximate likelihood ratio test with the Shimodaira-Hasegawa-like procedure (SH-aLRT) in PhyML, and SH-aLRT support values ≥0.90 were considered significant (Guindon et al. 2010). The Subtype/CRF-resolved phylogeny was visualized using FigTree v1.4.4 (https://github.com/rambaut/figtree/releases). Unique recombinant forms (URFs) were characterised by boot-scan analysis in SimPlot (Lole et al. 1999; Pineda-Peña et al. 2013; Struck et al. 2014).

### 2.5 Cluster analysis

Sequences were grouped into subtype-specific datasets and the most similar non-Kenyan sequences for each available Kenyan sequence were determined by a BLAST, as previously described (Esbjörnsson et al. 2016; Nazziwa et al. 2020; Nduva et al. 2020). Redundant sequences or clonal sequences from the same individual were removed from the dataset. All sequences were aligned by subtype and subtype-specific, and alignments were manually edited to exclude codon positions associated with drug resistance. Maximum-likelihood phylogenies were reconstructed in PhyML (Guindon et al. 2010). For each subtype, monophyletic clades with aLRT-SH support ≥0.9 and which were dominated (≥80 per cent) by Kenyan sequences (compared to reference sequences) were defined as Kenyan HIV-1 (Esbjörnsson et al. 2016; Hassan et al. 2017; Nazziwa et al. 2020; Nduva et al. 2020). Identified clusters were classified into dyads (2 sequences), networks (3–14 sequences), or large clusters (>14 sequences) (Esbjörnsson et al. 2016).

### 2.6 Bayesian phylodynamic inference

HIV-1 evolutionary origins and past population dynamics were determined using subsets of the main subtypes as well as for the large clusters identified in the cluster analysis. Only sequences with information on sampling dates were included in this analysis. The temporal signal was assessed in TempEst (v1.5.3) (Rambaut et al. 2016). Bayesian inferences were done in BEAST 1.10.4 using the Bayesian Skygrid model with an uncorrelated lognormal relaxed clock and inferred under the GTR + Γ4 + Ι substitution model (Drummond et al. 2005; Baele et al. 2012; Gill et al. 2013; Suchard et al. 2018). To enhance precision in estimating evolutionary parameters within and between clusters from different risk groups, a previously described hierarchical phylogenetic model (HPM) was specified on evolutionary parameters (Suchard et al. 2003). Each MCMC chain was run for 300 million states, sampling every 30,000th iteration and discarding the first 10 per cent as burn-in. Convergence was determined in Tracer v.1.7.0 and defined as effective sample sizes (ESS) ≥200 (Suchard et al. 2018)—and where this was not achieved, the burnin was adjusted or the analysis re-run with a longer chain (Hall, Woolhouse, and Rambaut 2016).

### 2.7 Bayesian phylogeographic inference

We computed a discrete phylogeographic inference using an empirical tree distribution—where the expected number of HIV-1 migrations for every pathway were inferred on a branch-by-branch basis as previously described (Lemey et al. 2009; Faria et al. 2014). Sampling province and risk group were used as independent discrete states. The asymmetric continuous-time Markov chain (CTMC) model was preferentially used as it relaxes the assumption of constant diffusion rates through time to realistically model phylogeographic processes (Lemey et al. 2009; Edwards et al. 2011). A robust counting approach implemented in BEAST was used to estimate the forward and reverse HIV-1 movement events (Markov jumps) between locations and risk group states along the branches of time dated phylogenetic trees (Minin and Suchard 2008). Well-supported movements and Bayes factors (BF) assessing statistical support were summarized using SPREAD v1.0.7, (BF ≥ 3 was considered significant) (Lemey et al. 2009). Maximum clade credibility (MCC) trees annotated with key demographic and epidemiological data were summarized in Tree-Annotator v1.10.4 (BEAST suite) and visualized in Figtree (v1.4.4).

### 2.8 Sensitivity analysis

In Kenya, the vast majority (35 per cent) of people with HIV-1 are in Nyanza province, followed by Rift Valley (17 per cent), Nairobi (13 per cent), Western (9 per cent), Central (9 per cent), Eastern (9 per cent), Coast (7 per cent), North Eastern (<1 per cent)—and modes of transmission estimates have shown that 64 per cent of infections result from heterosexual contact among casual or married couples, female sex work (14 per cent), men having sex with men (15 per cent) and injection drug use (4 per cent) (National AIDS and STI Control Programme 2017; Kenya National AIDS Control Council (NACC) 2018; National AIDS and STI Control Programme (NASCOP) 2020).

Phylogeographic analysis is sensitive to sampling size (on one hand, a small sample size might not be informative enough to infer migration profiles and on the other hand, analyzing thousands of sequences using the MCMC procedure is extremely computationally intensive and MCMC parameters often fail to converge) (Lemey et al. 2009; Faria et al. 2014; Bbosa et al. 2019). In addition, skewed sampling may further bias inference due to over-sampling some states compared to others. It is therefore essential that the sampling strategy ensures a sufficiently representative number of samples from each discrete state to avoid over-scoring transitions or counts in the empirical tree distribution. This necessitates down-sampling over-sampled states to reduce bias, and excluding under-represented states from the analysis (de Silva, Ferguson, and Fraser 2012; Volz 2012; Hall, Woolhouse, and Rambaut 2016). In our dataset, Western, Central, Eastern and North-Eastern provinces were underrepresented and hence excluded, and temporal focus was limited to sequences collected after 2004. Focus was on transitions between four locations (Nyanza, Rift Valley, Nairobi, and Coast), and between risk groups (MSM, PWID, FSW, and HET), and several approaches were used to limit sampling bias arising from the disproportional allocation of sequences per discrete state (described in detail below). HIV-1 sequences were first annotated with the year of sampling (2004–2019) and a discrete trait (risk group or location). In-house Perl scripts were used to randomize and select a set of sequences with uniform or proportional probability whilst also ensuring temporal sampling fidelity (Hall, Woolhouse, and Rambaut 2016).

In detail, in the first scenario, location-annotated HIV-1 sequences were sub-sampled proportional to the HIV-1 prevalence per geographic province. This procedure was independently replicated 30 times—resulting in 30 datasets each having 892 sequences of which 35 per cent were from Nyanza, 17 per cent Rift Valley, 13 per cent Nairobi, and 7 per cent Coast. A similar approach was taken with risk group as a discrete state—resulting in thirty datasets each having 802 sequences of which 64 per cent were from HET, 14 per cent FSW, 15 per cent MSM, and 4 per cent PWID. Cluster analysis (as described above) was performed independently for each dataset. Clusters having >14 sequences were identified—and discrete state phylogeographic analysis with Markov jumps inferences were then performed independently for each of the identified clusters.

Next, we further explored whether the population dynamics seen in recent years (i.e. 2010–2019) were different from those observed in the complete dataset (i.e. 2004–2019). In the second sensitivity analysis, HIV-1 A1 sequences collected during 2010–2019 were sub-sampled proportionally as was done in the first scenario—resulting in five independent datasets with location-annotation (each having 144 sequences—35 per cent from Nyanza, 17 per cent Rift Valley, 13 per cent Nairobi, and 7 per cent Coast), and five independent datasets with risk group annotation (each having 97 sequences—64 per cent HET, 14 per cent FSW, 15 per cent MSM, and 4 per cent PWID). However, unlike in the cluster-wise approach, the complete sub-sampled datasets were used directly to estimate virus migration between states. In the third sensitivity analysis, HIV-1 A1 sequences collected during 2010–2019 were sub-sampled uniformly into five datasets with equal number of sequences per discrete state. The location-annotated dataset had 100 sequences (25 sequences from each province), while the dataset annotated for risk group had 108 sequences (27 sequences for each risk group).

### 2.9 Statistical analysis

Changes in the proportion of HIV-1 subtypes and recombinants over time were assessed using the *nptrend* non-parametric test for trends (Cuzick 1985). Frequencies and percentages were used to describe the distribution of sequences within the study population. A logistic regression model was used to assess associations between individual sequence characteristics (e.g. subtype/CRF, location of sampling, risk group, and year [range] of sampling) and phylogenetic clustering. Variables with *p *< 0.1 in exploratory bivariable analyses were included in the multivariable model, in which *p *< 0.05 was defined as statistically significant. A Kruskal-Wallis H test and a post hoc Dunn’s test with Bonferroni correction for multiple comparisons were conducted to determine differences in HIV-1 evolutionary rate, cluster growth rates, and time to the most recent common ancestor (tMRCA) estimates among clusters from multiple risk groups. Statistics and summary plots were done using Stata 15 (StataCorp LLC, College Station, Texas, USA) and RStudio (version 1.2.5001) with the packages: *yarrr, circlize* and *ggplot2* (Gu et al. 2014; Wickham 2016; Phillips 2017).

### 2.10 Ethical considerations

All research was performed following relevant guidelines/regulations. For the newly generated sequences, informed consent for use of plasma samples was obtained from all participants from respective studies. Since published sequences were obtained from an open-access public domain, informed consent was not retrospectively obtained. Instead, we sought approval through a study protocol that was reviewed by the Kenya Medical Research Institute (KEMRI) Scientific and Ethics Review Unit (SERU 3547).

### 2.11 Data availability

Newly generated nucleotide sequences were deposited in GenBank under the following accession numbers: MT084914-MT085076, and OM109695-OM110282.

## 3. Results

### 3.1 Study population and sequence dataset

**Figure 1.**
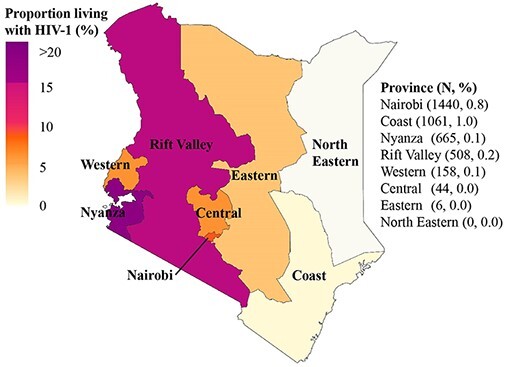
Map of Kenya highlighting geographic locations and sampling density. Map of Kenya highlighting geographic locations (former administrative provinces), HIV-1 burden per province (proportion of people with HIV-1 as per province in Kenya (Kenya National AIDS Control Council (NACC) 2018; Kenya National Bureau of Statistics 2019; National AIDS and STI Control Programme (NASCOP) 2019, 2020), and the sampling density (number of people with HIV-1 included in the study based on the estimated number of people with HIV-1 in Kenya).

We analysed 4058 HIV-1 *pol* sequences collected 1986–2019, of which 3303 (81.4 per cent) were previously published and 755 (18.6 per cent) newly generated for this study (Table 1, Supplementary Figure S1, and Supplementary Table S1). Most sequences were from HET (N = 3401, 83.8 per cent), followed by MSM (N = 372, 9.2 per cent), FSW (N = 227, 5.6 per cent), and PWID (N = 58, 1.4 per cent). Overall, these numbers represent an estimated sampling density of 0.3 per cent of the HIV-1 epidemic in Kenya, and specific sampling densities of 10.8 per cent for MSM, 1.7 per cent for PWID, 0.6 per cent for FSW, and 0.3 per cent for HET (Supplementary Table S2). Sequences were available from seven (of eight) former administrative provinces in Kenya: Nairobi (N = 1440, 35.5 per cent of the sequences in this study); Coast (N = 1061, 26.2 per cent); Nyanza (N = 665, 16.4 per cent); Rift Valley (N = 508, 12.5 per cent); Western (N = 158, 3.8 per cent); Central (N = 44, 1.1 per cent); Eastern (N = 6, 0.2 per cent); and 176 (4.3 per cent) sequences with missing data on sampling location (Table 1, and Fig. 1). All PWID sequences were derived from the Coast province. Sampling year and place were missing for 176 (4.3 per cent) of the newly generated HET sequences. These sequences were included in the assessment of subtype diversity in Kenya but excluded from the Bayesian phylodynamic analysis (which necessitates information on sampling date). In our dataset, 14 MSM identified as transgender persons. Subsequent sub-analyses were made to tease out clustering patterns specific for transgender persons relative to other risk groups.

**Table 1.** Demographics and distribution of newly generated and published Kenyan HIV-1 pol sequences by risk group.

	Risk group					
Category		HET	MSM	FSW	PWID	Total
Sequences	Published	2987 (87.8%)	159 (42.7%)	99 (43.6%)	58 (100.0%)	3303 (81.4%)
	New	414 (12.2%)	213 (57.3%)	128 (56.4%)	0 (0.0%)	755 (18.6%)
Province	Nairobi	1212 (35.6%)	137 (36.8%)	91 (40.1%)	0 (0.0%)	1440 (35.5%)
	Coast	704 (20.7%)	178 (47.9%)	121 (53.3%)	58 (100.0%)	1061 (26.2%)
	Nyanza	594 (17.5%)	57 (15.3%)	14 (6.2%)	0 (0.0%)	665 (16.4%)
	Rift Valley	507 (14.9%)	0 (0.0%)	1 (0.4%)	0 (0.0%)	508 (12.5%)
	Western	158 (4.7%)	0 (0.0%)	0 (0.0%)	0 (0.0%)	158 (3.9%)
	Central	44 (1.3%)	0 (0.0%)	0 (0.0%)	0 (0.0%)	44 (1.1%)
	Eastern	6 (0.2%)	0 (0.0%)	0 (0.0%)	0 (0.0%)	6 (0.2%)
	Missing^a^	176 (5.2%)	0 (0.0%)	0 (0.0%)	0 (0.0%)	176 (4.3%)
Year (range)	2001–2010	2077 (64.4%)	118 (31.7%)	170 (74.9%)	58 (100.0%)	2423 (59.7%)
	2011–2019	1070 (33.2%)	254 (68.3%)	36 (15.9%)	0 (0.0%)	1360 (33.5%)
	1986–2000	78 (2.4%)	0 (0.0%)	21 (9.3%)	0 (0.0%)	99 (2.4%)
	Missing^a^	176 (5.2%%)	0 (0.0%)	1 (0.0%)	2 (0.0%)	176 (4.3%)
Total		3401 (83.8%)	372 (9.2%)	227 (5.6%)	58 (1.4%)	4058 (100.0%)

Abbreviations: MSM, men who have sex with men; PWID, people who inject drugs; FSW, female sex worker; HET, at-risk men and women who did not report sex work or male same-sex behaviour.

a Sequences lacking information on year and geographic area of sampling.

### 3.2 HIV-1 sub-subtype A1 and subtype D dominated the epidemic in Kenya

**Figure 2.**
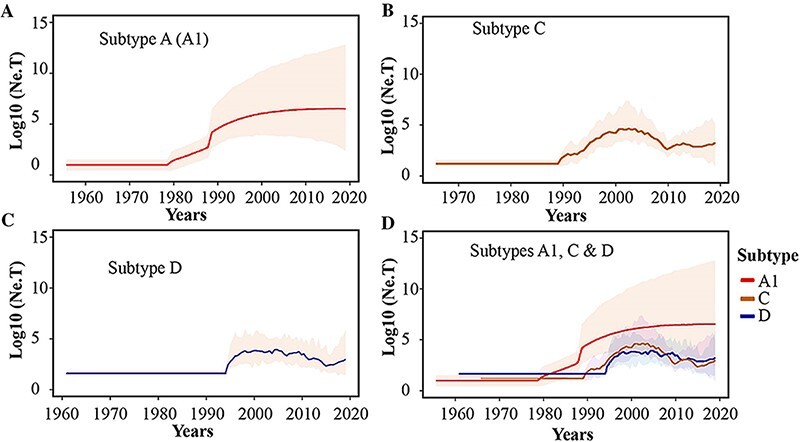
Population dynamics of HIV-1 sub-subtype A1, subtype D and subtype C lineages in Kenya. Bayesian Skygrid plots showing effective population size of the (A) HIV-1 sub-subtype A1, (B) HIV-1 subtype C and (C) HIV-1 subtype D lineages in the Kenyan dataset. Median estimates of the effective population size overtime are shown as a continuous line in each plot (coloured Red for sub-subtype A1, Brown for subtype C, and Blue for subtype D). The shaded area represents the 95 per cent higher posterior density intervals of the inferred effective population size for each lineage.

Among the combined new and published Kenyan sequences (N = 4058, Supplementary Table S3), HIV-1 sub-subtype A1 was most common (70.5 per cent) followed by subtype D (11.4 per cent, Supplementary Figure S2). Sub-subtype A1 was also the most common HIV-1 strain in all provinces and amongst all risk groups (Supplementary Table S4, and Supplementary Table S5, respectively). Temporal trend analysis in subtype distribution was restricted to the period after 2004 that comprised 92.0 per cent of the sequences (Supplementary Figure S2). Sub-subtype A1 infections increased from 59.7 per cent to 78.3 per cent, 2004–2019 (p < 0.001). No significant change was seen for subtype C (p = 0.30) or subtype D (p = 0.59), whereas subtype G decreased from 1.2 per cent to 0.0 per cent, 2004–2019 (p = 0.013). Overall, CRFs decreased from 2.7 per cent to 0.0 per cent, 2004–2019 (p = 0.005), whereas URFs decreased from 11 per cent to 0.9 per cent, 2004–2019 (p = 0.001). Bayesian inference also revealed that the effective population size estimates for HIV-1 sub-subtype A1 were consistently higher than those for HIV-1 subtypes C and D throughout the study period (Fig. 2).

### 3.3 HIV-1 geographic mixing within and between provinces in Kenyan

Overall, 1832 (45 per cent) of Kenyan sequences were found in 409 clusters including sub-subtype A1 (N = 306, 74.8 per cent), subtype C (N = 25, 6.1 per cent), and subtype D (N = 78, 19.1 per cent) clusters (Table 2, Supplementary Table S6, Supplementary Figures S3 and S4).

**Table 2.** Kenyan HIV-1 clusters (N=409) grouped into different subtypes and HIV-1 transmission routes.

	Dyads^a^	Networks^b^	Large clusters^c^	Total (N,%)
Subtype				
A (A1)	182 (59%)	105 (34%)	19 (6%)	306 (75%)
C	16 (64%)	8 (32%)	1 (4%)	25 (6%)
D	51 (65%)	27 (35%)	0 (0%)	78 (19%)
Risk category				
HET	204 (65%)	101 (32%)	11 (3%)	316 (77%)
Mixed^d^	24 (51%)	16 (34%)	7 (15%)	47 (11%)
MSM	13 (35%)	23 (62%)	1 (3%)	37 (9%)
FSW	7 (100.0%)	0 (0%)	0 (0%)	7 (2%)
PWID	1 (50%)	0 (0%)	1 (50%)	2 (<1%)
Total	249 (61%)	140 (34%)	20 (5%)	409

Abbreviations: HET, heterosexual transmission; Mixed; MSM, men who have sex with men; FSW, female sex work; PWID, people who inject drugs. *Risk groups in mixed clusters (N, proportion of mixed clusters): MSM/HET (15, 32%), FSW/HET (15, 32%), MSM/FSW/HET (9, 19%), MSM/FSW (6, 13%), MSM/PWID/FSW/HET (1, 2%), and PWID/HET (1, 2%).

^a^Dyads: clusters of 2 sequences ^b^Networks: clusters of 3–14 sequences ^c^Large clusters: clusters of >14 sequences. ^d^Clusters with sequences from multiple risk groups.

Overall, 1485 (51.9 per cent) of sub-subtype A1 sequences, 137 (48.1 per cent) subtype C, and 210 (45.6 per cent) subtype D formed clusters. The remaining 1375 (48.1 per cent) sub-subtype A1, 148 (51.9 per cent) subtype C, and 251 (54.5 per cent) sequences were singletons (Supplementary Table S6). Majority (N = 248, 60.6 per cent) were province-exclusive, including clusters from Nairobi (N = 107, 26.2 per cent), Coast (N = 58, 14.2 per cent), Nyanza (N = 51, 12.5 per cent), Rift Valley (N = 23, 5.6 per cent), Western (N = 6, 1.5 per cent), and Central (N = 3, 0.7 per cent). The remaining clusters (N = 161, 39.4 per cent) were mixed between different geographic provinces (Supplementary Figure S5a).

### 3.4 Within-risk group clustering dominated among Kenyan HIV-1 clusters

Majority (N = 362, 88.5 per cent) of the clusters represented within-risk group HIV-1 transmission including HET (N = 316; 72.1 per cent), MSM (N = 37, 9.1 per cent), FSW (N = 7, 1.7 per cent) and PWID (N = 2, 0.5 per cent). Further and amongst PWID, only two clusters were identified (one dyad and one large cluster, both PWID exclusive), with the large cluster comprising 80 per cent of all PWID sequences in the dataset (N = 41). The remaining clusters (N = 47, 11.5 per cent) involved mixed linkages between different risk groups including MSM/HET (N = 15, 3.7 per cent of all clusters), FSW/HET (N = 15, 3.7 per cent), MSM/FSW/HET (N = 9, 2.2 per cent), MSM/FSW (N = 6, 1.5 per cent), MSM/PWID/FSW/HET (N = 1, 0.2 per cent), and PWID/HET (N = 1, 0.2 per cent) mixed clusters (Table 2, Supplementary Figure S5b). A sub-analysis of clustering patterns involving transgender people showed that nine of 14 (64.3 per cent) clustered with MSM, four clustered with HET (28.6 per cent), and one did not cluster with any other sequences in the dataset (7.1 per cent). Compared to HET, MSM and PWID sequences were more likely to cluster (adjusted odds ratio [aOR] 4.4, 95 per cent confidence interval [CI] 3.2–6.0, p < 0.001; and aOR 3.4, CI 1.8–6.5, p < 0.001, respectively, Table 3).

**Table 3.** Factors associated with clustering among HIV-1 sequences from Kenya.

		Bivariate analysis^a^		Multivariate analysis	
Characteristics		OR (95% CI)	p-value	aOR (95% CI)	p-value
Risk category	HET	Reference			
	MSM	3.8 (3–4.8)	<0.001	4.4 (3.2–6.0)	<0.001
	PWID	4.7 (2.5–8.8)	<0.001	3.4 (1.8–6.5)	<0.001
	FSW	0.6 (0.5–0.9)	0.003	1.2 (0.8–1.7)	0.391
Subtype	A1	Reference			
	C	0.9 (0.7–1.1)	0.215		
	D	0.8 (0.6–0.9)	0.011	0.68 (0.6–0.9)	<0.001
Year (range)	1986–2000	Reference		Reference	
	2001–2010	3.7 (2.2–6.2)	<0.001	3.9 (2.1–7.0)	<0.001
	2011–2019	5.1 (3.0–8.7)	<0.001	5.3 (2.9–9.9)	<0.001
Province	Central	Reference			
	Coast	1.3 (0.7–2.4)	0.383		
	Eastern	0.3 (0–3)	0.314		
	Nairobi	1.6 (0.9–2.9)	0.141		
	Nyanza	1.4 (0.7–2.6)	0.297		
	Rift Valley	0.8 (0.4–1.6)	0.576		
	Western	1 (0.5–1.9)	0.936		
	Missing^a^	1 (0.5–1.9)	0.945		
Sequence category	New	Reference			
	Published	1.2 (1.1–1.5)	0.007	0.6 (0.5–0.8)	<0.001

Abbreviations: MSM, men who have sex with men; PWID, people who inject drugs; FSW, female sex worker; HET, at-risk men and women who did not report sex work or male same-sex behaviour.

a Only variables with a p <0.1 in the bivariate analysis were included in the multivariate model (thus subtype C and province were excluded from the multivariate analysis).

### 3.5 The effective population size has stabilised over time amongst all risk groups

**Figure 3.**
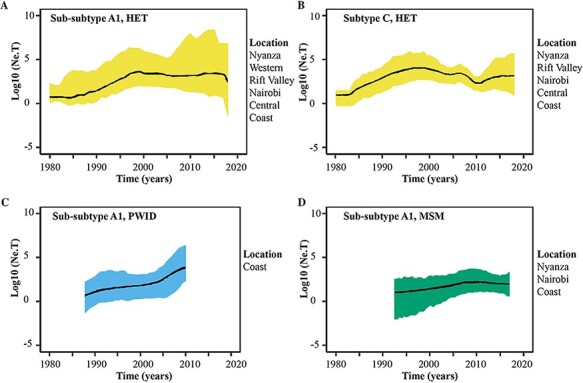
HIV-1 risk group-specific estimates in the effective population size through time in Kenya. Bayesian Skygrid plots showing historical population dynamics of (A) the main HIV-1 sub-subtype A1 HET clusters, (B) the only large subtype C HET cluster, (C) the only large HIV-1 sub-subtype A1 PWID cluster and (D) the only large HIV-1 sub-subtype A1 MSM cluster in Kenya. Median estimates of the number of individuals contributing to new infections over time are shown as a continuous line coloured as per the dominant risk group per cluster (bluish-green: MSM; sky blue: PWID; and yellow: HET). The area shaded grey represents the 95 per cent higher posterior density intervals of the inferred effective population size. Information on geographic representation per cluster is provided in the figure legends.

The correlation between divergence from root and time of sampling was low in our dataset (i.e. R^2^ = 0.139, 0.136, and 0.121 for the sub-subtype A1, subtype C, and subtype D datasets, respectively, Supplementary Figure S6). Thus normal priors were specified for the time of the most recent common ancestor (tMRCA) of sub-subtype A1, subtype C and subtype D, based on previous estimations (Faria et al. 2014, 2019). The inference of HIV-1 dynamics in the Kenyan epidemic was based on a Bayesian phylodynamic analysis of the large Kenyan HIV-1 clusters (19 sub-subtype A1 and one subtype C cluster (Supplementary Table S7). All sub-subtype A1 HET clusters exhibited similar dynamics (Supplementary Figure S7) and were merged in one plot to assess overall dynamics among HET (Fig. 3A). The number of effective infections (proportional to the transmission rate over the prevalence) for HET increased over time from 1987 to the mid-2000s, after which infections stabilised. The number of Kenyan PWID contributing to new HIV-1 infections over time increased gradually from 1987 to 2010, the latest sampling date for PWID (Fig. 3C), whereas the MSM-exclusive cluster showed stable dynamics with no periods of exponential growth between 1991 and 2019, the latest sampling date for MSM (Fig. 3D). The only large subtype C cluster that was found was a HET cluster—this cluster showed similar dynamics as the sub-subtype A1 HET clusters, with increasing effective population size from 1983 to the early 2000s followed by a stabilisation (Fig. 3B).

### 3.6 Evolutionary parameters were similar among clusters of different risk groups

**Figure 4.**
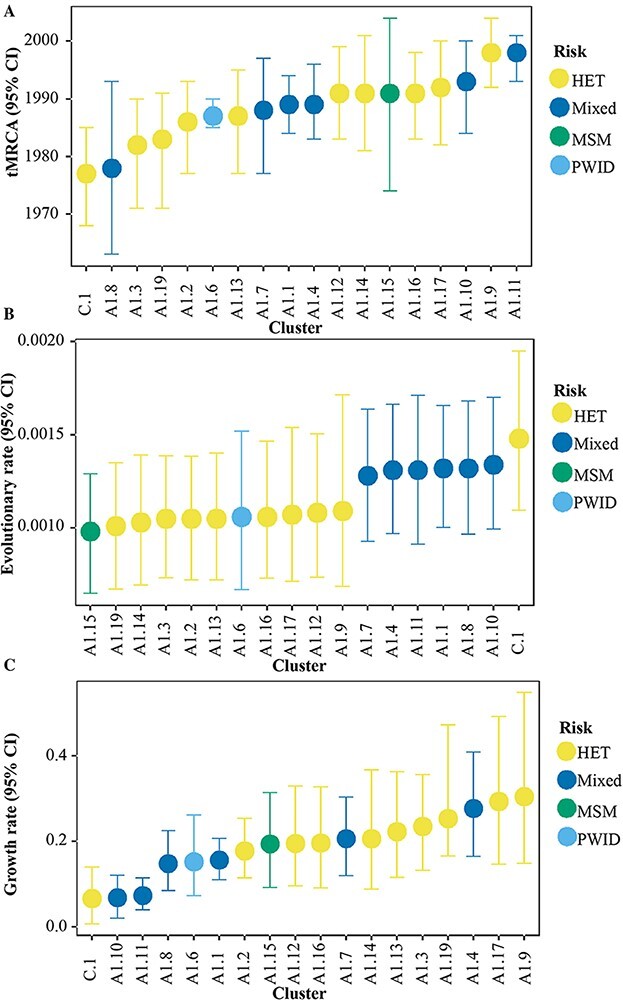
Date of origin, evolutionary rate, and growth rate among sub-subtype A1 and subtype C clusters of different risk groups. Time to the most recent common ancestor (A), evolutionary rate (B), and growth rate (C) estimates among seventeen sub-subtype A1 and one subtype C clusters. Median estimates and 95 per cent higher posterior density interval are shown for the different categories per cluster, coloured by the dominant risk group per cluster. Results are not shown for two clusters (A1.5.HET and A1.18.HET) whose parameters did not converge.

Subtype C had the earliest tMRCA (1977, 95 per cent higher posterior density [HPD] interval: 1968–1985) of all clusters. The median tMRCA estimates of sub-subtype A1 clusters indicated multiple introductions into Kenya over 42 years (1978–2019), with most clusters introduced between the late 1980s and early 1990s. The earliest tMRCA for a Kenyan HET cluster was estimated to 1978 (95 per cent HPD interval: 1971–1990); MSM to 1991 (HPD interval: 1974–2004); and PWID to 1987 (HPD interval: 1985–1990). The median HIV-1 evolutionary rates ranged from 1.01 × 10^−3^ to 1.3 × 10^−3^ substitutions site^−1^ year^−1^ (s/s/y) for subtype A1 in HET clusters and 1.28 × 10^−3^ to 1.34 × 10^−3^ s/s/y for mixed-risk group clusters. The median HIV-1 evolutionary rate for the only large MSM cluster was 9.80 × 10^−4^ s/s/y, and 1.06 × 10^−3^ s/s/y for the only large PWID cluster. Pairwise comparison of median evolutionary rates (with Bonferroni correction for multiple comparisons) showed no difference in evolutionary rates between HET and MSM (p = 0.169), HET and PWID (p = 1.00), and MSM and PWID (p = 0.297). No statistical differences were found between tMRCA estimates or cluster growths between clusters of different risk groups, respectively (p = 0.822, and p = 0.321, Table 4, Fig. 4).

**Table 4.** Estimated dates of origin and evolutionary parameters of the large Kenyan HIV-1 clusters.

Cluster	tMRCA^a^	Evolutionary rate (E^−3^)	Growth rate (per year)
A1.1.MIX	1989 [1984, 1994]	1.32 [1.00, 1.66]	0.16 [0.11, 0.21]
A1.2.HET	1986 [1977, 1993]	1.05 [0.73, 1.39]	0.18 [0.12, 0.25]
A1.3.HET	1982 [1971, 1990]	1.05 [0.72, 1.39]	0.24 [0.13, 0.36]
A1.4.MIX	1989 [1983, 1996]	1.31 [0.97, 1.67]	0.28 [0.17, 0.41]
A1.6.PWID	1987 [1985, 1990]	1.06 [0.67, 1.52]	0.15 [0.07, 0.26]
A1.7.MIX	1988 [1977, 1997]	1.28 [0.93, 1.64]	0.21 [0.12, 0.30]
A1.8.MIX	1978 [1963, 1993]	1.32 [0.97, 1.69]	0.15 [0.09, 0.23]
A1.9.HET	1998 [1992, 2004]	1.09 [0.69, 1.71]	0.31 [0.15, 0.55]
A1.10.MIX	1993 [1984, 2000]	1.34 [0.99, 1.70]	0.07 [0.02, 0.12]
A1.11.HET	1998 [1993, 2001]	1.31 [0.91, 1.71]	0.07 [0.04, 0.12]
A1.12.HET	1991 [1983, 1999]	1.08 [0.73, 1.50]	0.19 [0.10, 0.33]
A1.13.HET	1987 [1977, 1995]	1.05 [0.72, 1.40]	0.22 [0.12, 0.36]
A1.14.HET	1991 [1981, 2001]	1.03 [0.69, 1.39]	0.21 [0.09, 0.37]
A1.15.MSM	1991 [1974, 2004]	0.98 [0.65, 1.29]	0.19 [0.09, 0.31]
A1.16.HET	1991 [1983, 1998]	1.06 [0.73, 1.47]	0.19 [0.09, 0.33]
A1.17.HET	1992 [1982, 2000]	1.07 [0.71, 1.54]	0.29 [0.15, 0.49]
A1.19.HET	1983 [1971, 1991]	1.01 [0.67, 1.35]	0.25 [0.17, 0.47]
C.1.HET	1977 [1968, 1985]	1.48 [1.09, 1.95]	0.07 [0.01, 0.14]

Abbreviations: HET, Heterosexual transmission; Mixed; MSM, men who have sex with men; FSW, female sex work; MTMC, perinatal transmission; PWID, people who inject drugs. Results are not shown for two clusters (A1.5.HET and A1.18.HET) whose parameters did not converge.

a HPD: Higher posterior density interval. TMRCA: time to the most recent common ancestor. Data are median and 95% higher posterior density intervals.

### 3.7 Evidence of West-to-East HIV-1 migration, and transmission from HET to key populations

**Figure 5.**
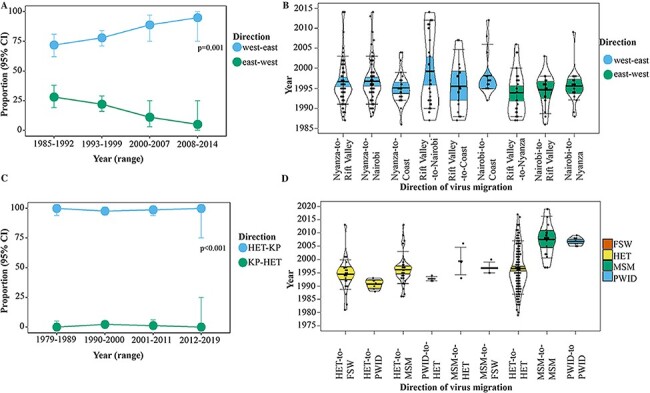
Proportion and dates of HIV-1 transitions between geographic provinces and risk groups. Dates of HIV-1 transitions between geographic provinces and risk groups summarised from trait-annotated maximum clade credibility trees. Plots represent (A) proportion of West-to-East vs East-to-West geographic migration over time, (B) dates of HIV-1 dissemination between different geographic locations (where group median and interquartile range are coloured by the direction of transmission—coloured sky blue: West-to-East, and vermillion: East-to-West), (C) proportion of HIV-1 transmission from heterosexuals to key populations and vice-versa over time, and (D) dates of HIV-1 transmission within and between different risk groups (where group median and interquartile range are coloured by ‘source’ risk group—coloured green: MSM; sky blue: PWID; vermillion: FSW; yellow: HET). Only transitions with a posterior probability higher than 0.90 are plotted. Dots in the pirate plots represent HIV-1 migration events.

Phylogeographic analysis was based on HIV-1 sub-subtype A1—the strain with the highest number of sequences in our study, and the most dominant strain circulating strain in Kenya. In all sensitivity analyses, Western, Central and Eastern provinces were excluded as they had the smallest number of sequences in the study, and sequences from transgender people and MSM were analysed together as one risk group. The Markov jumps estimates from the cluster-wise phylogeographic inference indicated that the majority (62.6 per cent) of HIV-1 jumps occurred within Kenyan borders whilst the remaining involved HIV-1 export (24.1 per cent) from Kenya to other countries, and HIV-1 import (13.2 per cent) to Kenya (Table 5). The proportion of West-to-East jumps over time was significantly higher than that of East-to-West jumps (p = 0.001, Figs 5A and 4B). West-to-East migration accounted for the majority (76.1 per cent) of all within-country jumps—including jumps from Nyanza to Nairobi (10.3 per cent), Rift Valley to Nairobi (9.8 per cent), Nyanza to Rift Valley (9.2 per cent), Nyanza to Coast (6.3 per cent), Rift Valley to Coast (6.3 per cent), and Nairobi to Coast (5.7 per cent). East-to-West migration accounted for only 23.9 per cent within-country jumps and comprised jumps from Rift Valley to Nyanza (7.5 per cent), Nairobi to Nyanza (4.6 per cent), and Nairobi to Rift Valley (2.9 per cent, Fig. 5B). Pairs of geographic provinces located next to each other were involved in an extensive cyclic HIV-1 exchange—including transmission from Nyanza to Rift Valley (9.2 per cent forward jumps versus 7.5 per cent reverse jumps) and Rift Valley to Nairobi (9.8 per cent vs 2.9 per cent). Although Coast province received a significant proportion of translocated HIV-1 lineages (18.3 per cent of all HIV-1 jumps), no within-country HIV-1 jumps were observed as originating from Coast province. Uniform and proportional sub-sampling of the sequences collected 2010–2019 indicated more West-to-East virus flow than vice-versa (p < 0.001 for all comparisons, Table 6, Supplementary Figures S8a and S8b).

**Table 5.** Number of expected (Markov) jumps (BF ≥3) inferred for HIV-1 migration between geographic locations and between risk groups based on the cluster-wise sub-sampling approach.

The direction of migration events (from-to)	Number of HIV-1 jumps (N, %)
Geographic	174 (100.0%)
Within-country	109 (62.6%)
Nyanza-Nairobi	18 (10.3%)
Rift Valley-Nairobi	17 (9.8%)
Nyanza-Rift Valley	16 (9.2%)
Rift Valley-Nyanza	13 (7.5%)
Nyanza-Coast	11 (6.3%)
Rift Valley-Coast	11 (6.3%)
Nairobi-Coast	10 (5.7%)
Nairobi-Nyanza	8 (4.6%)
Nairobi-Rift Valley	5 (2.9%)
Export from Kenya	42 (24.1%)
Nyanza-Ref	20 (11.5%)
Rift Valley-Ref	13 (7.5%)
Nairobi-Ref	6 (3.4%)
Coast-Ref	3 (1.7%)
Import into Kenya	23 (13.2%)
Ref-Coast	9 (5.2%)
Ref-Nyanza	5 (2.9%)
Ref-Rift Valley	5 (2.9%)
Ref-Nairobi	4 (2.3%)
Risk group	47 (100.0%)
HET-FSW	16 (34.0%)
HET-MSM	15 (31.9%)
HET-PWID	8 (17.0%)
PWID-HET	3 (6.4%)
MSM-HET	3 (6.4%)
MSM-FSW	2 (4.3%)

Abbreviations: Ref, reference HIV-1 *pol* sequences from the global epidemic that clustered closely with Kenyan sequences; HET, heterosexual transmission; Mixed; MSM, men who have sex with men; FSW, female sex work; MTMC, perinatal transmission; PWID, people who inject drugs.

**Table 6.** The number of HIV-1 jumps (2010–2019) based on proportional and uniform sub-sampling.

Jumps direction (from-to)	Number of Jumps (N)	
Jumps between locations	Proportional sub-sampling	Uniform sub-sampling
West to East	319 (88%)	213 (78%)
Nyanza-Rift Valley	129 (36%)	50 (18%)
Nyanza-Nairobi	113 (31%)	73 (27%)
Nyanza-Coast	50 (14%)	54 (20%)
Nairobi-Coast	8 (2%)	19 (7%)
Rift Valley-Nairobi	14 (4%)	8 (3%)
Rift Valley-Coast	5 (1%)	9 (3%)
East to west	43 (12%)	61 (22%)
Rift Valley-Nyanza	11 (3%)	6 (2%)
Nairobi-Rift Valley	9 (2%)	21 (8%)
Nairobi-Nyanza	9 (2%)	25 (9%)
Coast-Nyanza	7 (2%)	3 (1%)
Coast-Nairobi	4 (1%)	3 (1%)
Coast-Rift Valley	3 (1%)	3 (1%)
Jumps between risk groups		
HET to key populations	126 (94%)	126 (72%)
HET-FSW	64 (48%)	75 (43%)
HET-MSM	58 (43%)	46 (26%)
HET-PWID	4 (3%)	5 (3%)
Key populations to HET	3 (2%)	20 (11%)
FSW-HET	1 (1%)	15 (9%)
PWID-HET	1 (1%)	3 (2%)
MSM-HET	1 (1%)	2 (1%)
Key populations to others	5 (4%)	29 (17%)
FSW-MSM	2 (1%)	14 (8%)
FSW-PWID	1 (1%)	4 (2%)
MSM-FSW	2 (1%)	9 (5%)
MSM-PWID	0 (0%)	1 (1%)
PWID-FSW	0 (0%)	1 (1%)
PWID-MSM	0 (0%)	0 (0%)

Abbreviations: HET, heterosexual; MSM, men who have sex with men; FSW, female sex workers; PWID, people who inject drugs.

The cluster-wise phylogeographic inference showed that 82.9 per cent of virus jumps between risk groups were from HET (involving HET-to-FSW [34.0 per cent], HET-to-MSM [31.9 per cent], and HET-to-PWID [17.0 per cent]). Only 12.8 per cent virus jumps were from key populations (involving MSM-to-HET [6.4 per cent] and PWID-to-HET [6.4 per cent], Fig. 5D). The remaining were MSM-to-FSW virus jumps (4.3 per cent, Table 5). Also, the proportion of virus jumps from HET to key populations over time was significantly higher compared with virus jumps from key populations to HET (p < 0.001, Fig. 5C). The earliest estimated Markov jump event from HET-to-FSW occurred in 1981, followed by HET-to-MSM (1986), and HET-to-PWID (1990, Fig. 5D). Virus jumps among HET were common as early as during the 1980s while virus jumps among MSM (i.e. MSM-to-MSM) and among PWID (i.e. PWID-to-PWID) increased during the 1990s and 2000s, respectively (Fig. 5D). Uniform and proportional sub-sampling of the sequences collected 2010–2019 indicated more HIV-1 jumps from HET to key populations than vice-versa (p < 0.001 for all comparisons, Table 6, Supplementary Figures S8c and S8d).

## 4. Discussion

We show that HIV-1 transmission in Kenya was largely compartmentalized by risk groups. This result is based on the identification of 409 statistically supported phylogenetic clusters—where a majority (88.5 per cent) represents within-risk group clustering. Furthermore, we found that 11.5 per cent of the clusters represented HIV-1 mixing between risk groups—including approximately 7.6 per cent HIV-1 mixing between MSM and HET in Kenya. These findings are consistent with previous phylogenetic data in Coastal Kenya demonstrating minimal HIV-1 mixing between key populations and the heterosexual population (Bezemer et al. 2014; Nduva et al. 2020). We have previously estimated frequent (85 per cent) within-risk group clustering, and minimal (15 per cent) HIV-1 mixing between MSM and the HET in Coastal Kenya (Nduva et al. 2020). Likewise, Bezemer and colleagues—albeit with a small sample size and sequences only from Nairobi and Coast province only found one HIV-1 MSM/HET link, indicating infrequent HIV-1 mixing between MSM and HET (Bezemer et al. 2014). The phylogeographic inference indicated a higher proportion of HIV-1 jumps from HET to MSM, FSW and PWID. However, the detected virus jumps represent rare events as overall transmission between risk groups is itself rare in the Kenyan epidemic (as shown in the cluster analysis). Also, majority of HIV-1 jumps from HET to other risk groups occurred in the more distant past—and likely represent the historical flow of HIV-1 from HET to other risk groups. Overall, our findings indicate that contrary to concerns by the Ministry of Health in Kenya (National AIDS and STI Control Programme 2017), HIV-1 key populations may not disproportionately transmit HIV-1 to heterosexuals in the general epidemic. It is well established that the vast majority of HIV-1 transmission in Kenya could be attributed to risky heterosexual behaviours (Kenya National AIDS Control Council 2009; Gouws and Cuchi 2012).

Overall, our study highlights important dynamics in HIV-1 spread in the context of a mixed HIV-1 epidemic and support the hypothesis of frequent within-risk group transmission and limited between-risk group transmission (Bezemer et al. 2014; Nduva et al. 2020). This hypothesis is further strengthened by findings from a review of 35 studies assessing HIV-1 mixing between HIV-1 populations in sub-Saharan Africa highlighting the predominance of within-risk group transmission chains in most countries (Nduva et al. 2021). To reduce population-level HIV-1 incidence in sub-Saharan Africa, HIV-1 control programs may require both broad-reaching interventions aimed at the general epidemic, as well as strengthening micro-strategies that address disparities among population categories (including scale-up of ART, HIV-1 testing and other prevention programs directed towards key populations such as MSM, PWID and FSW who are most-at-risk of infection) (Cremin et al. 2017; Kelly et al. 2018; Koss et al. 2021; Smith et al. 2021).

In this study, HIV-1 transmission in Kenya involved predominantly West-to-East dissemination, notably from high HIV-1 prevalence regions (including the former Nyanza province in Western Kenya) to comparatively lower HIV-1 prevalence regions (including former Coastal province). Irrespective of transmission risk, the largest number of people with HIV-1, and approximately 40 per cent of all newly diagnosed HIV-1 infections have been suggested to occur in Western Kenya (National AIDS and STI Control Programme (NASCOP) 2020). It is therefore plausible that the observed HIV-1 dissemination pattern reflects considerable HIV-1 transmission from high-to-low HIV-1 prevalence regions, a finding that likely applies to other sub-Saharan African countries with substantial within-country variation in the prevalence of HIV-1. However, our findings contrast data from Uganda showing significant virus flow from low-to-high HIV-1 prevalence populations along the Lake Victoria (Bbosa et al. 2019; Grabowski et al. 2020; Ratmann et al. 2020). In the current study, we did not have data on fishing folk and we did not assess transmission between fishing folk and inland communities. Yet, it is possible that some undisclosed fishing-folk were grouped with HET (unless where the risk group was known) and classified as belonging to the Nyanza province. The gradient in HIV-1 prevalence in Kenya decreases Eastwards, and we observe an overall higher proportion of HIV-1 migration from provinces in the West (Nyanza and Rift valley) towards provinces in the East (such as the Coast province). Mathematical modelling and empirical evidence have shown that directed approaches may reduce HIV-1 incidence across sub-Saharan Africa (Gerberry et al. 2014; Mcgillen et al. 2016; Grabowski et al. 2017; Vandormael et al. 2019). Optimizing existing prevention strategies in geographic HIV-1 hotspots (Dwyer-Lindgren et al. 2019) in sub-Saharan Africa (such as Western Kenya) may therefore result in declining population-level HIV-1 incidence (Bailey et al. 2007; Anderson et al. 2014).

Our study represents one of the largest national-level analyses of HIV-1 *pol* diversity that has been done in Africa. However, we were still limited by a low sampling density and data on how the study participants in the published studies were identified for sequencing. Low sampling likely resulted in missing links in identified Kenyan clusters and low probability of detecting some rare subtypes circulating in Kenya (Novitsky et al. 2014). Moreover, PWID and their partners, as well as the clients of sex workers, were less likely to get into treatment studies and were therefore underrepresented in this study. It is therefore likely that the rates of HIV-1 transmissions from FSW, MSM and PWID to the HET population were underestimated owing to those missing links. Despite the lower sampling density of HET compared to MSM, PWID, and FSW sequences in the full dataset, our sensitivity analyses controlling for sampling bias indicated more virus jumps from HET to key populations. Yet, majority of these jumps may not reflect current transmission dynamics between risk groups as they might occurred in the distant past. Also, excluding some geographic locations from our sensitivity analysis due to few numbers of sequences from these provinces in our dataset may have resulted in missing transmission chains and could bias phylogeographic estimates of the geographic HIV-1 spread in Kenya (Novitsky et al. 2014; Hassan et al. 2017). Nonetheless, the excluded provinces have HIV-1 prevalence rates lower than the national average and based on findings from this analysis, it is unlikely that they would be major sources of HIV-1 in Kenya. Lastly, we assessed HIV-1 flow between populations, not between individuals, and these population-level inferences may not be extrapolated to individual transmissions. Also, virus jumps between risk populations in the phylogeographic analyses may not be equated with transmission events because the discrete phylogeographic modelling used in this analysis only accounts for between-risk group jump, and not within-risk group jumps. Other similar studies from developed settings with concentrated epidemics and dense sampling among infected individuals (as well as readily available patient demographic data) have provided information useful in HIV-1 prevention (Fisher et al. 2010; Kouyos et al. 2010; Volz et al. 2013; Poon et al. 2016; Ratmann et al. 2016; Sallam et al. 2017; Ragonnet-Cronin et al. 2018; Vasylyeva et al. 2018). To minimise phylogenetic uncertainties arising from low sample coverage, future studies in sub-Saharan Africa should aim to achieve higher sampling densities and aim to include sequences collected in years that are more recent to determine more active Kenyan clusters.

In conclusion, we have estimated the rates of transmission between the general heterosexual population and HIV-1 key populations, and between geographic regions with varying HIV-1 prevalence in Kenya. We showed that high HIV-1 prevalence regions may be important sources of HIV-1 to lower-prevalence regions, and that the Kenyan HIV-1 epidemic is largely compartmentalized by risk groups and that the contribution of key populations to the wider heterosexual transmission network may be significantly lower than vice versa. In the mixed Kenyan HIV-1 epidemic, targeting HIV-1 key populations needs to occur concurrently with strengthening broad interventions in the general population. These findings could pave the way towards strengthening HIV-1 control in Kenya and other countries in sub-Saharan Africa.

## Supplementary Material

veac016_Supp
