# Supplementary material for: Quantifying rates of HIV-1 flow between risk groups and geographic locations in Kenya: A country-wide phylogenetic study
Source: Virus Evol. 2022 Mar 3;8(1):veac016. doi: 10.1093/ve/veac016 (PMC8962731; doi:10.1093/ve/veac016)
Supplement: veac016_Supp [file veac016_supp.zip › Nduva-23-02-2022-Supplementary.docx]

**SUPPLEMENTARY DATA**

**Files in this Data Supplement:**

**Supplementary tables**

Table S1. The number and sources of plasma samples used to generate new HIV-1 pol sequences

Table S2. The number of Kenyan HIV-1 partial *pol* sequences (N=4058, 1986-2019) sequences analysed in this study compared to national estimates of the number of people living with HIV-1 in Kenya belonging to different risk groups and geographic regions.

Table S3. The number and temporal distribution of Kenyan HIV-1 partial *pol* sequences (N=4058, 1986-2019).

Table S4. Distribution of HIV-1 subtypes by geographic area.

Table S5. Distribution of HIV-1 subtypes by risk groups.

Table S6. Proportions of Kenyan sequences in clusters relative to Kenyan sequences that did not cluster and their distribution into risk groups and geographic provinces.

Table S7. Characteristics of large Kenyan clusters (N=20) used in the inference of past population dynamics.

**Supplementary figures.**

Figure S1. A summary scheme of sampling criteria in this study.

Figure S2. Distribution of HIV-1 sequences and subtypes (1986-2019).

Figure S3. Maximum-likelihood trees used to identify transmission clusters.

Figure S4. Size and subtype distribution of 409 Kenyan HIV-1 clusters identified in this study.

Figure S5. Graphical summary of the distribution of 409 Kenyan clusters by geographic locations and risk groups.

Figure S6. Root-to-tip regression analyses of phylogenetic temporal signal.

Figure S7. Population dynamics in the HET and mixed-risk group HIV-1 clusters.

Figure S8. Pirate plots quantifying direction and number of HIV-1 jumps between geographic locations and risk groups.

**Tables**

**Table S1.** **A summary scheme of sampling criteria in this study.**

| **Site** | **Risk group** | | | |
| --- | --- | --- | --- | --- |
|  | **HET** | **MSM** | **FSW** | **PWID** |
| **KWTRP** | 48 | 21 | 107 | 0 |
| **NHRS** | 0 | 57 | 14 | 0 |
| **KAVI-ICR** | 30 | 0 | 7 | 0 |
| **SWOP** | 0 | 50 | 0 | 0 |
| **TRANSFORM** | 0 | 85 | 0 | 0 |
| **KEMRI-CGHR** | 336 | 0 | 0 | 0 |
| **Total** | 414 | 213 | 128 | 0 |

Abbreviations: HET, heterosexual adults; MSM, men who have sex with men; FSW, female sex workers; PWID, people who inject drugs. Site abbreviations: KWTRP, Kenya Medical Research Institute (KEMRI) -Wellcome Trust (Coastal Kenya); NHRS, Nyanza Reproductive Health Society (in Western Kenya); KAVI-ICR, Kenya AIDS Vaccine Initiative’s Institute of Clinical Research (in Nairobi, Central Kenya); SWOP, Sex Workers Outreach Program clinics in Nairobi, TRANSFORM, a cohort of transfeminine people and cisgender men who have sex with men in Nairobi; KEMRI-CGHR, Kenya Medical Research Institute (KEMRI) – Centre for Global Health Research (Western Kenya).

**Table S2.** **The number of Kenyan HIV-1 partial *pol* sequences (N=4058, 1986-2019) analysed in this study compared to national estimates of the number of people with HIV-1 in Kenya belonging to different risk groups and geographic regions.**

| **Characteristic** |  | **^a^Total population estimates** | ^b^**PWHIV (N)** | ^c^**Sample size (N)** | ^d^**Sampling density (%)** |
| --- | --- | --- | --- | --- | --- |
| **Overall** | Kenya | 47,564,296 | 1,493,413 (100%) | 4,058 | 0.3 |
| **Sampling location** | Nyanza | 7,163,260 | 526,972 (35%) | 665 | 0.1 |
|  | Rift Valley | 12,752,966 | 247,127 (17%) | 508 | 0.2 |
|  | Nairobi | 4,397,073 | 190,993 (13%) | 1,440 | 0.8 |
|  | Western | 4,128,162 | 141,561 (9%) | 158 | 0.1 |
|  | Central | 5,482,239 | 141,306 (9%) | 44 | 0.0 |
|  | Eastern | 6,821,049 | 132,232 (9%) | 6 | 0.0 |
|  | Coast | 4,329,474 | 108,994 (7%) | 1,061 | 1.0 |
|  | North Eastern | 2,490,073 | 4,196 (<1%) | 0 | 0.0 |
| **Risk group** | HET | 26,642,987 | 1,341,164 (90%) | 3,401 | 0.3 |
|  | FSW | 133,675 | 40,103 (3%) | 227 | 0.6 |
|  | MSM | 19,175 | 3,452 (>1%) | 372 | 10.8 |
|  | PWID | 18,327 | 3,482 (>1%) | 58 | 1.7 |
|  | Children (>15 years) | 2,0750,132 | 105,213 (7%) | 0 | 0.0 |

Abbreviations: HET, heterosexual adults; MSM, men who have sex with men; FSW, female sex workers; PWID, people who inject drugs; PWHIV, people with HIV. ^a^Kenyan population estimates as of 2019 (Kenya National Bureau of Statistics, 2019). ^b^The estimated number of PWHIV as per geographic area [computed from national population data (Kenya National Bureau of Statistics, 2019) and HIV-1 prevalence data per geographic region (National AIDS and STI Control Programme (NASCOP), 2020)], and risk groups [computed from key populations estimates (National AIDS and STI Control Programme (NASCOP), 2019) and HIV-1 prevalence per risk group (Kenya National AIDS control council (NACC), 2018)] in Kenya. ^c^The number of people living with HIV-1 included in the study, and ^d^the estimated proportion of people living with HIV-1 in Kenya included in the study.

**Table S3. The number and temporal distribution of Kenyan HIV-1 partial *pol* sequences (N=4058, 1986-2019).**

|  | **Risk group** |  |  |  | **Province** |  |  |  |  |  |  |  |
| --- | --- | --- | --- | --- | --- | --- | --- | --- | --- | --- | --- | --- |
| **Sampling year** | **HET** | **MSM** | **FSW** | **PWID** | **Nairobi** | **Coast** | **Nyanza** | **Rift Valley** | **Western** | **Central** | **Eastern** | **Total** |
| **1986** | 0 | 0 | 2 | 0 | 2 | 0 | 0 | 0 | 0 | 0 | 0 | **2** |
| **1991** | 4 | 0 | 0 | 0 | 4 | 0 | 0 | 0 | 0 | 0 | 0 | **4** |
| **1993** | 1 | 0 | 0 | 0 | 1 | 0 | 0 | 0 | 0 | 0 | 0 | **1** |
| **1994** | 0 | 0 | 1 | 0 | 1 | 0 | 0 | 0 | 0 | 0 | 0 | **1** |
| **1996** | 4 | 0 | 12 | 0 | 0 | 12 | 4 | 0 | 0 | 0 | 0 | **16** |
| **1997** | 13 | 0 | 6 | 0 | 6 | 0 | 13 | 0 | 0 | 0 | 0 | **19** |
| **1998** | 7 | 0 | 0 | 0 | 0 | 0 | 7 | 0 | 0 | 0 | 0 | **7** |
| **1999** | 18 | 0 | 0 | 0 | 4 | 0 | 13 | 1 | 0 | 0 | 0 | **18** |
| **2000** | 31 | 0 | 0 | 0 | 7 | 8 | 8 | 8 | 0 | 0 | 0 | **31** |
| **2001** | 8 | 0 | 5 | 0 | 11 | 1 | 0 | 1 | 0 | 0 | 0 | **13** |
| **2002** | 4 | 0 | 7 | 0 | 11 | 0 | 0 | 0 | 0 | 0 | 0 | **11** |
| **2003** | 3 | 0 | 0 | 0 | 0 | 2 | 0 | 1 | 0 | 0 | 0 | **3** |
| **2004** | 199 | 0 | 0 | 0 | 5 | 0 | 171 | 23 | 0 | 0 | 0 | **199** |
| **2005** | 198 | 0 | 10 | 0 | 44 | 18 | 125 | 21 | 0 | 0 | 0 | **208** |
| **2006** | 380 | 28 | 20 | 0 | 154 | 57 | 16 | 201 | 0 | 0 | 0 | **428** |
| **2007** | 554 | 12 | 35 | 0 | 282 | 293 | 26 | 0 | 0 | 0 | 0 | **601** |
| **2008** | 236 | 19 | 75 | 0 | 97 | 230 | 3 | 0 | 0 | 0 | 0 | **330** |
| **2009** | 228 | 33 | 16 | 0 | 61 | 173 | 0 | 43 | 0 | 0 | 0 | **277** |
| **2010** | 267 | 26 | 2 | 58 | 127 | 118 | 107 | 1 | 0 | 0 | 0 | **353** |
| **2011** | 243 | 13 | 2 | 0 | 96 | 26 | 6 | 129 | 1 | 0 | 0 | **258** |
| **2012** | 284 | 6 | 4 | 0 | 144 | 6 | 5 | 0 | 139 | 0 | 0 | **294** |
| **2013** | 201 | 5 | 0 | 0 | 137 | 31 | 2 | 36 | 0 | 0 | 0 | **206** |
| **2014** | 94 | 6 | 2 | 0 | 36 | 11 | 55 | 0 | 0 | 0 | 0 | **102** |
| **2015** | 58 | 22 | 2 | 0 | 25 | 5 | 19 | 0 | 0 | 33 | 0 | **82** |
| **2016** | 44 | 68 | 22 | 0 | 37 | 40 | 50 | 2 | 3 | 1 | 1 | **134** |
| **2017** | 56 | 118 | 4 | 0 | 126 | 14 | 17 | 8 | 8 | 5 | 0 | **178** |
| **2018** | 90 | 2 | 0 | 0 | 22 | 2 | 18 | 33 | 7 | 5 | 5 | **92** |
| **2019** | 0 | 14 | 0 | 0 | 0 | 14 | 0 | 0 | 0 | 0 | 0 | **14** |
| ***Missing** | 176 | 0 | 0 | 0 | 0 | 0 | 0 | 0 | 0 | 0 | 0 | **176** |
| **Total** | 3,401 | 372 | 227 | 58 | 1,440 | 1,061 | 665 | 508 | 158 | 44 | 6 | **4,058** |

Abbreviations: HET, heterosexual; MSM, men who have sex with men; FSW, female sex work; PWID, people who inject drugs.

^*^Missing: some (N=176, 4% of all sequences, all HET) of the newly generated sequences lacked data on the geographic area of sampling.

**Table S4. Distribution of HIV-1 subtypes by geographic provinces in Kenya.**

| **Subtype (N, %)** | **Central** | **Coast** | **Eastern** | **Nairobi** | **Nyanza** | **Rift Valley** | **Western** | **Unknown** | **Total** |
| --- | --- | --- | --- | --- | --- | --- | --- | --- | --- |
| **A1** | 35 (1.2%) | 765 (26.8%) | 5 (0.2%) | 1044 (36.5%) | 424 (14.8%) | 304 (10.6%) | 118 (4.1%) | 165 (5.8%) | 2860 (70.5%) |
| **B** | 0 (0.0%) | 0 (0.0%) | 0 (0.0%) | 1 (100.0%) | 0 (0.0%) | 0 (0.0%) | 0 (0.0%) | 0 (0.0%) | 1 (0.0%) |
| **C** | 2 (0.7%) | 75 (26.3%) | 0 (0.0%) | 84 (29.5%) | 53 (18.6%) | 55 (19.3%) | 10 (3.5%) | 6 (2.1%) | 285 (7.0%) |
| **CRF01_AE** | 0 (0.0%) | 0 (0.0%) | 0 (0.0%) | 1 (100.0%) | 0 (0.0%) | 0 (0.0%) | 0 (0.0%) | 0 (0.0%) | 1 (0.0%) |
| **CRF02_AG** | 0 (0.0%) | 1 (100.0%) | 0 (0.0%) | 0 (0.0%) | 0 (0.0%) | 0 (0.0%) | 0 (0.0%) | 0 (0.0%) | 1 (0.0%) |
| **CRF10_CD** | 0 (0.0%) | 8 (32%) | 0 (0.0%) | 7 (28%) | 4 (16%) | 2 (8%) | 4 (16%) | 0 (0.0%) | 25 (0.6%) |
| **CRF16_A2D** | 3 (7%) | 9 (20.9%) | 0 (0.0%) | 19 (44.2%) | 10 (23.3%) | 2 (4.7%) | 0 (0.0%) | 0 (0.0%) | 43 (1.1%) |
| **CRF18_cpx** | 0 (0.0%) | 0 (0.0%) | 0 (0.0%) | 1 (100.0%) | 0 (0.0%) | 0 (0.0%) | 0 (0.0%) | 0 (0.0%) | 1 (0.0%) |
| **CRF21_A2D** | 0 (0.0%) | 8 (38.1%) | 0 (0.0%) | 6 (28.6%) | 4 (19.1%) | 2 (9.5%) | 1 (4.8%) | 0 (0.0%) | 21 (0.5%) |
| **CRF43_02G** | 0 (0.0%) | 0 (0.0%) | 0 (0.0%) | 0 (0.0%) | 1 (100.0%) | 0 (0.0%) | 0 (0.0%) | 0 (0.0%) | 1 (0.0%) |
| **D** | 3 (0.7%) | 86 (18.7%) | 1 (0.2%) | 169 (36.7%) | 84 (18.2%) | 100 (21.7%) | 13 (2.8%) | 5 (1.1%) | 461 (11.4%) |
| **G** | 0 (0.0%) | 4 (20%) | 0 (0.0%) | 8 (40%) | 3 (15%) | 5 (25%) | 0 (0.0%) | 0 (0.0%) | 20 (0.5%) |
| **URF** | 1 (0.3%) | 105 (31.1%) | 0 (0.0%) | 100 (29.6%) | 82 (24.3%) | 38 (11.2%) | 12 (3.6%) | 0 (0.0%) | 338 (8.3%) |
| **Total** | 44 (1.1%) | 1061 (26.2%) | 6 (0.2%) | 1440 (35.5%) | 665 (16.4%) | 508 (12.5%) | 158 (3.8%) | 176 (4.3%) | 4058 (100.0%) |

Abbreviations: CRF, circulating recombinant form; URF, unique recombinant form; HET, heterosexual; MSM, men who have sex with men; FSW, female sex work; PWID, people who inject drugs. *Missing: some of the newly generated sequences (N=176, 4% of all sequences, all HET) had missing information on the geographic area of sampling.

**Table S5. Distribution of HIV-1 subtypes by risk groups in Kenya.**

| **Subtype** | **Risk group** |  |  |  |  |
| --- | --- | --- | --- | --- | --- |
|  | **HET** | **MSM** | **FSW** | **PWID** | **Total** |
| **A1** | 2388 (70.2%) | 276 (74.2%) | 140 (61.7%) | 56 (96.6%) | 2860 (70.5%) |
| **B** | 1 (0.0%) | 0 (0.0%) | 0 (0.0%) | 0 (0.0%) | 1 (0.0%) |
| **C** | 232 (6.8%) | 31 (8.3%) | 20 (8.8%) | 2 (3.4%) | 285 (7.0%) |
| **CRF01_AE** | 1 (0.0%) | 0 (0.0%) | 0 (0.0%) | 0 (0.0%) | 1 (0.0%) |
| **CRF02_AG** | 1 (0.0%) | 0 (0.0%) | 0 (0.0%) | 0 (0.0%) | 1 (0.0%) |
| **CRF10_CD** | 23 (0.7%) | 1 (0.3%) | 1 (0.4%) | 0 (0.0%) | 25 (0.6%) |
| **CRF16_A2D** | 36 (1.1%) | 4 (1.1%) | 3 (1.3%) | 0 (0.0%) | 43 (1.1%) |
| **CRF18_cpx** | 0 (0.0%) | 0 (0.0%) | 1 (0.4%) | 0 (0.0%) | 1 (0.0%) |
| **CRF21_A2D** | 16 (0.5%) | 1 (0.3%) | 4 (1.8%) | 0 (0.0%) | 21 (0.5%) |
| **CRF43_02G** | 1 (0.0%) | 0 (0.0%) | 0 (0.0%) | 0 (0.0%) | 1 (0.0%) |
| **D** | 391 (11.5%) | 49 (13.2%) | 21 (9.3%) | 0 (0.0%) | 461 (11.4%) |
| **G** | 18 (0.5%) | 0 (0.0%) | 2 (0.9%) | 0 (0.0%) | 20 (0.5%) |
| **URF** | 293 (8.6%) | 10 (2.7%) | 35 (15.4%) | 0 (0.0%) | 338 (8.3%) |
| **Total** | 3401 (100.0%) | 372 (100.0%) | 227 (100.0%) | 58 (100.0%) | 4058 (100.0%) |

Abbreviations: CRF, circulating recombinant form; URF, unique recombinant form; HET, heterosexual; MSM, men who have sex with men; FSW, female sex work; PWID, people who inject drugs. *Missing: some of the newly generated sequences (N=176, 4% of all sequences, all HET) had missing information on the geographic area of sampling.

**Table S6. Proportions of Kenyan sequences in clusters relative to Kenyan sequences that did not cluster and their distribution into subtype, risk groups and geographic provinces.**

|  | **Clustered (N, %)** | **Did not cluster (N, %)** | **Total (N, %)** |
| --- | --- | --- | --- |
| **Subtype** |  |  |  |
| A1 | 1485 (51.9%) | 1375 (48.1%) | 2860 (100.0%) |
| C | 137 (48.1%) | 148 (51.9%) | 285 (100.0%) |
| D | 210 (45.6%) | 251 (54.5%) | 461 (100.0%) |
| **Risk group** |  |  |  |
| HET | 1441 (42.4%) | 1960 (57.6%) | 3401 (100.0%) |
| MSM | 273 (73.4%) | 99 (26.6%) | 372 (100.0%) |
| FSW | 73 (32.2%) | 154 (67.8%) | 227 (100.0%) |
| PWID | 45 (77.6%) | 13 (22.4%) | 58 (100.0%) |
| **Sampling location** |  |  |  |
| Nairobi | 720 (50%) | 720 (50%) | 1440 (100.0%) |
| Coast | 481 (45.3%) | 580 (54.7%) | 1061 (100.0%) |
| Nyanza | 311 (46.8%) | 354 (53.2%) | 665 (100.0%) |
| Rift Valley | 175 (34.5%) | 333 (65.6%) | 508 (100.0%) |
| Western | 60 (38%) | 98 (62%) | 158 (100.0%) |
| Central | 17 (38.6%) | 27 (61.4%) | 44 (100.0%) |
| Eastern | 1 (16.7%) | 5 (83.3%) | 6 (100.0%) |
| *Missing | 67 (38.1%) | 109 (61.9%) | 176 (100.0%) |
| **Total** | 1832 (45.2%) | 2226 (54.9%) | 4058 (100.0%) |

Abbreviations: HET, heterosexual; MSM, men who have sex with men; FSW, female sex work; PWID, people who inject drugs.

*Missing information on the geographic area of sampling

**Table S7. Characteristics of large Kenyan clusters (N=20) used in the inference of past population dynamics.**

| **Cluster** | **1** | **2** | **3** | **4** | **5** | **6** | **7** | **8** | **9** | **10** | **11** | **12** | **13** | **14** | **15** | **16** | **17** | **18** | **19** | **20** |
| --- | --- | --- | --- | --- | --- | --- | --- | --- | --- | --- | --- | --- | --- | --- | --- | --- | --- | --- | --- | --- |
| **Subtype** | A1 | A1 | A1 | A1 | A1 | A1 | A1 | A1 | A1 | A1 | A1 | A1 | A1 | A1 | A1 | A1 | A1 | A1 | A1 | C |
| **Sampling location (%)** | | | | | | | | | | | | | | | | | | | | |
| Central | 1 | 0 | 0 | 0 | 0 | 0 | 14 | 0 | 4 | 4 | 0 | 0 | 0 | 0 | 0 | 0 | 6 | 0 | 0 | 4 |
| Coast | 31 | 29 | 26 | 47 | 3 | 100 | 24 | 30 | 4 | 29 | 4 | 14 | 29 | 33 | 29 | 15 | 63 | 60 | 13 | 28 |
| Nairobi | 31 | 44 | 15 | 32 | 60 | 0 | 38 | 48 | 38 | 39 | 96 | 36 | 29 | 28 | 52 | 30 | 19 | 20 | 53 | 30 |
| Nyanza | 25 | 15 | 41 | 18 | 0 | 0 | 14 | 0 | 21 | 18 | 0 | 23 | 17 | 11 | 14 | 30 | 13 | 7 | 0 | 19 |
| Ref | 0 | 0 | 0 | 0 | 0 | 0 | 0 | 0 | 0 | 0 | 0 | 0 | 17 | 6 | 5 | 5 | 0 | 13 | 20 | 5 |
| Rift Valley | 8 | 7 | 18 | 0 | 28 | 0 | 3 | 15 | 21 | 11 | 0 | 18 | 4 | 22 | 0 | 20 | 0 | 0 | 7 | 14 |
| Western | 3 | 4 | 0 | 3 | 10 | 0 | 7 | 6 | 13 | 0 | 0 | 9 | 4 | 0 | 0 | 0 | 0 | 0 | 7 | 0 |
| **Risk groups (%)** | | | | | | | | | | | | | | | | | | | | |
| FSW | 7 | 1 | 4 | 9 | 0 | 0 | 3 | 6 | 3 | 0 | 0 | 4 | 4 | 0 | 0 | 5 | 6 | 7 | 7 | 5 |
| HET | 81 | 95 | 96 | 83 | 100 | 0 | 92 | 84 | 93 | 42 | 97 | 92 | 96 | 94 | 0 | 95 | 94 | 80 | 93 | 87 |
| MSM | 12 | 4 | 0 | 9 | 0 | 0 | 6 | 11 | 3 | 58 | 0 | 4 | 0 | 6 | 100 | 0 | 0 | 7 | 0 | 3 |
| PWID | 0 | 0 | 0 | 0 | 0 | 100 | 0 | 0 | 0 | 0 | 4 | 0 | 0 | 0 | 0 | 0 | 0 | 6 | 0 | 5 |

Abbreviations: Ref, reference HIV-1 *pol* sequences from the global epidemic that clustered closely with Kenyan sequences; HET, heterosexual; MSM, men who have sex with men; FSW, female sex work; PWID, people who inject drugs.

**Supplementary figures**

**Figure S1. Study scheme**

A summary scheme of sampling criteria in this study.

**
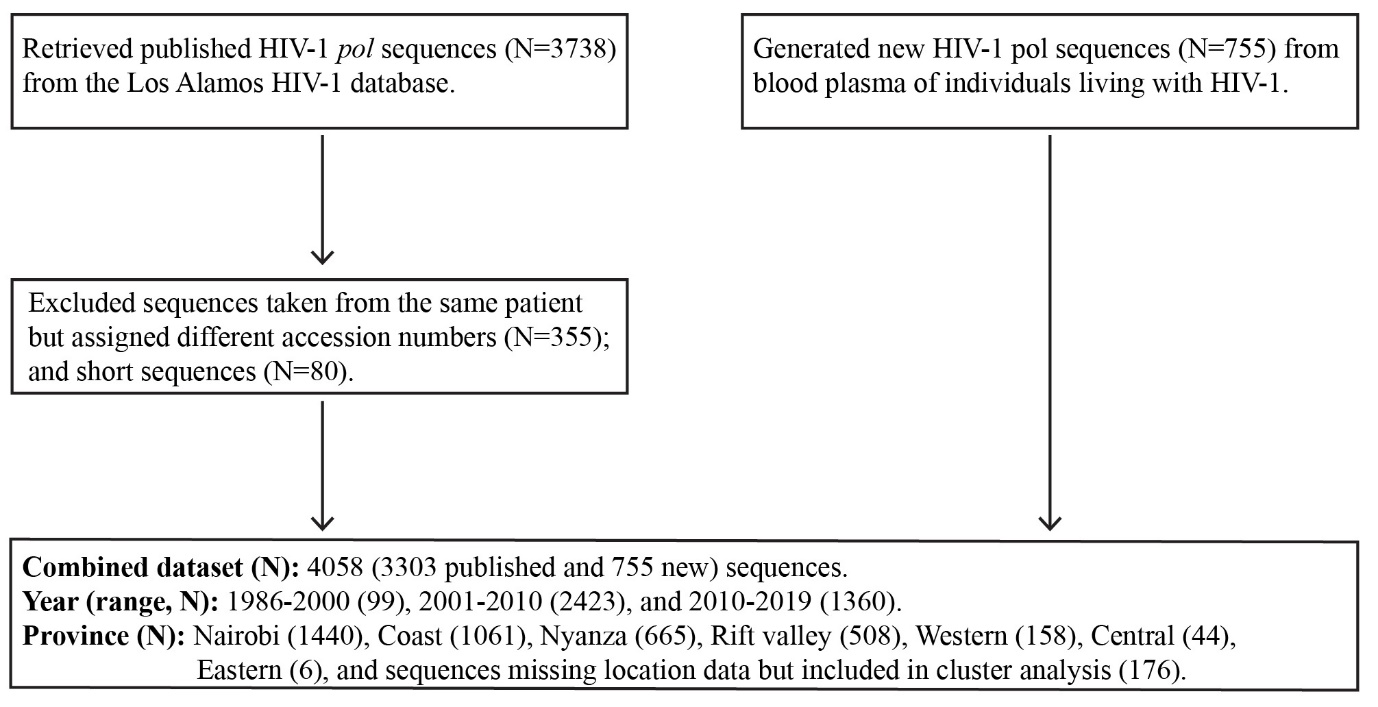
**

**Figure S2. Distribution of HIV-1 sequences and subtypes in this study (1986-2019).**

(**a**) Temporal distribution of the number of Kenyan HIV-1 sequences in this study. (**b**) ML phylogenetic reconstruction of HIV-1 group M genetic diversity based on genetic sequences (N=4058) from Kenya. Branch tips on the phylogenetic tree and proportion of HIV-1 lineages in different geographical locations are coloured according to subtypes (orange: sub-subtype A1; yellow: subtype B; brown: subtype C; blue: subtype D; maroon: subtype G; grey: circulating recombinant forms (CRFs); green: unique recombinant forms (URFs); black: HIV-1 group M reference sequences. (**c**) Temporal changes (2004-2019) in the overall proportion of HIV-1 subtypes and recombinants over two-years intervals in Kenya. A *p*<0.05 denotes a statistically significant increase or decrease in the proportion of respective circulating strains over time.


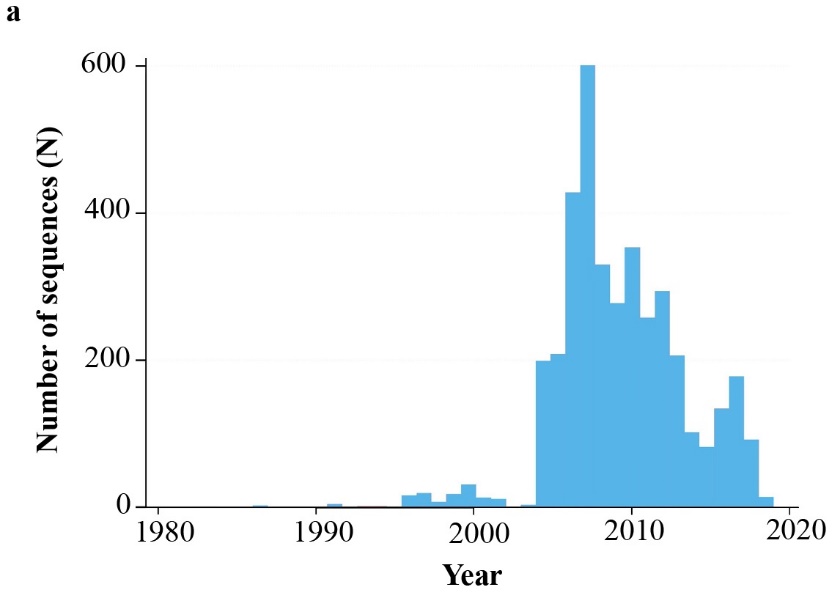


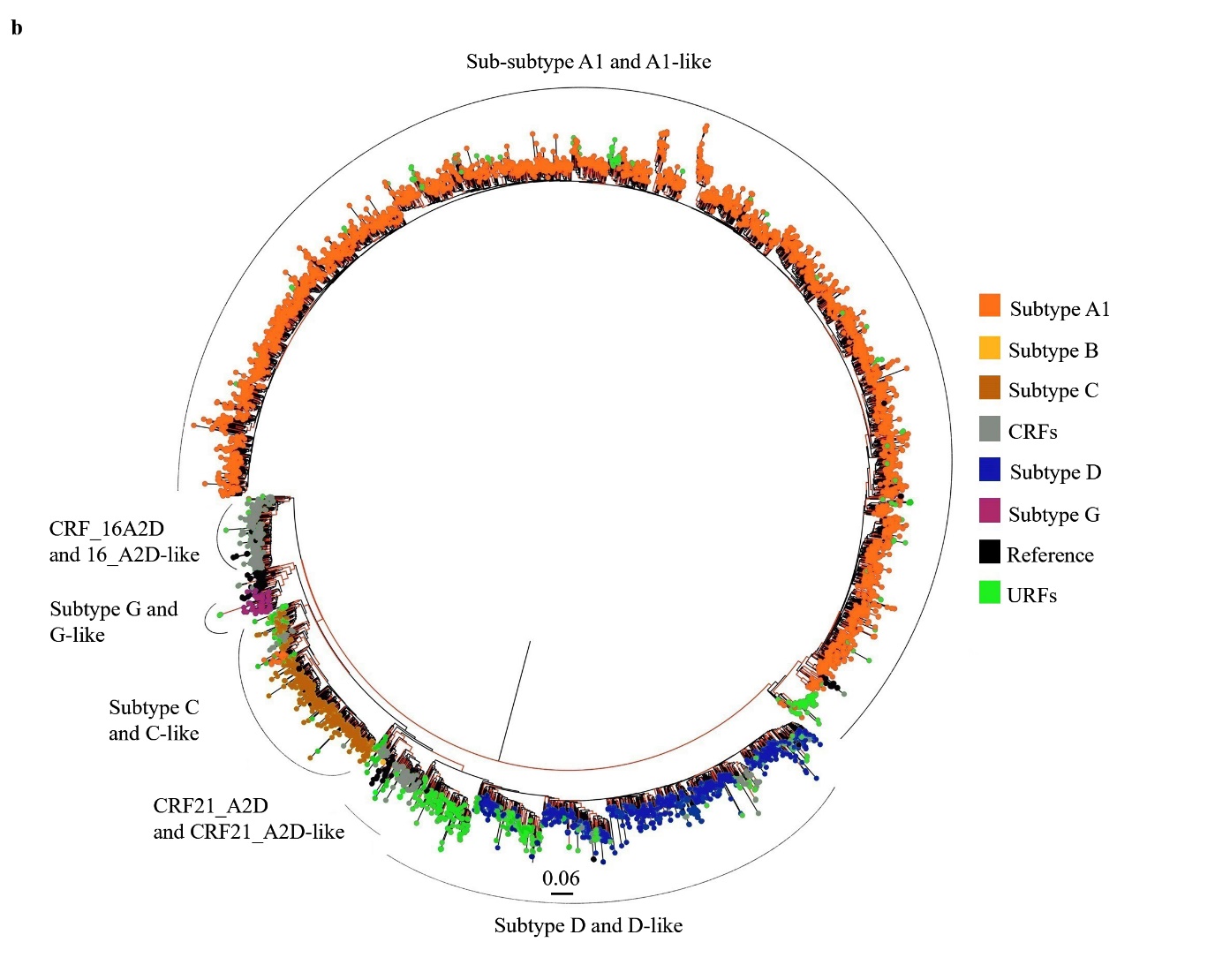


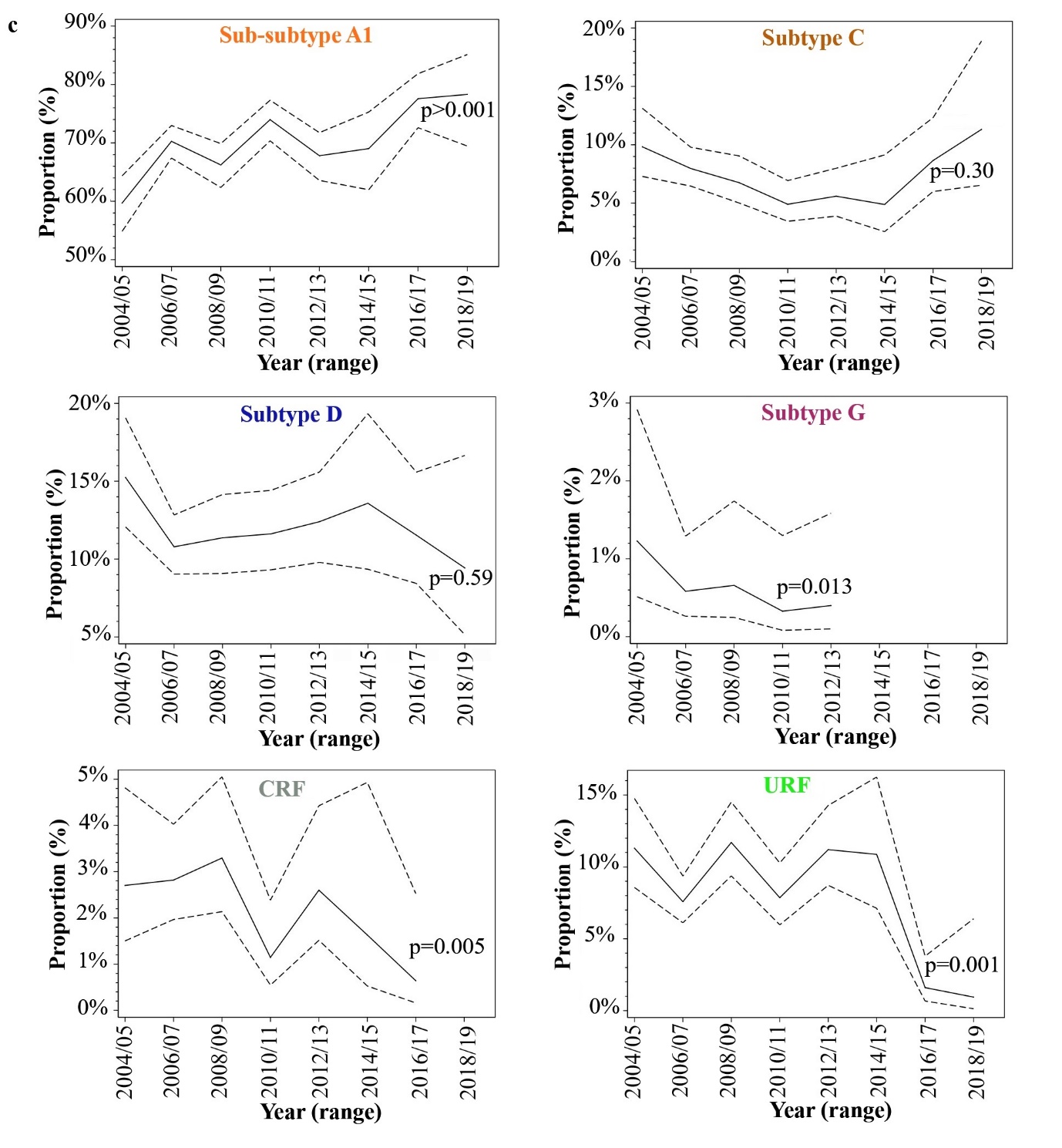


**Figure S3. Maximum-likelihood trees used to identify transmission clusters.**

Maximum-likelihood trees used for identification of Kenya HIV-1 clusters. Trees represent (**a**) sub-subtype A1, (**b**) subtype C, and (**c**) subtype D transmission clusters, respectively. Each phylogeny is rooted at the midpoint. Monophyletic clusters with SH-aLRT support ≥0.9 and which have ≥80% sequences from Kenya are highlighted in grey. To enhance cluster visualization, some branches containing either reference sequences or Kenyan sequences that did not clusters have been collapsed (shown as black triangles). Branch tips within respective clusters are coloured as per cluster risk group (green: MSM; sky blue: PWID; vermillion: FSW; yellow: HET; and black: Reference sequences). Red bars in the respective trees represent statistically supported branches (i.e. branches with SH-aLRT support ≥0.9). Scale bars represent the genetic distance in substitutions per site in all phylogenies.

**
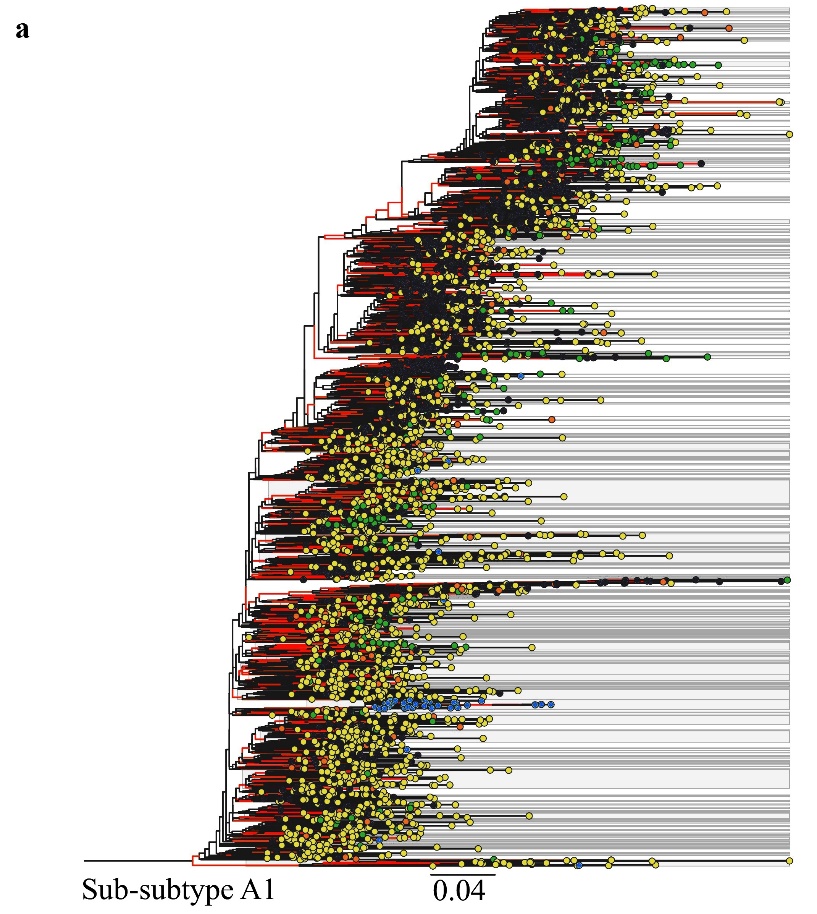
**

**
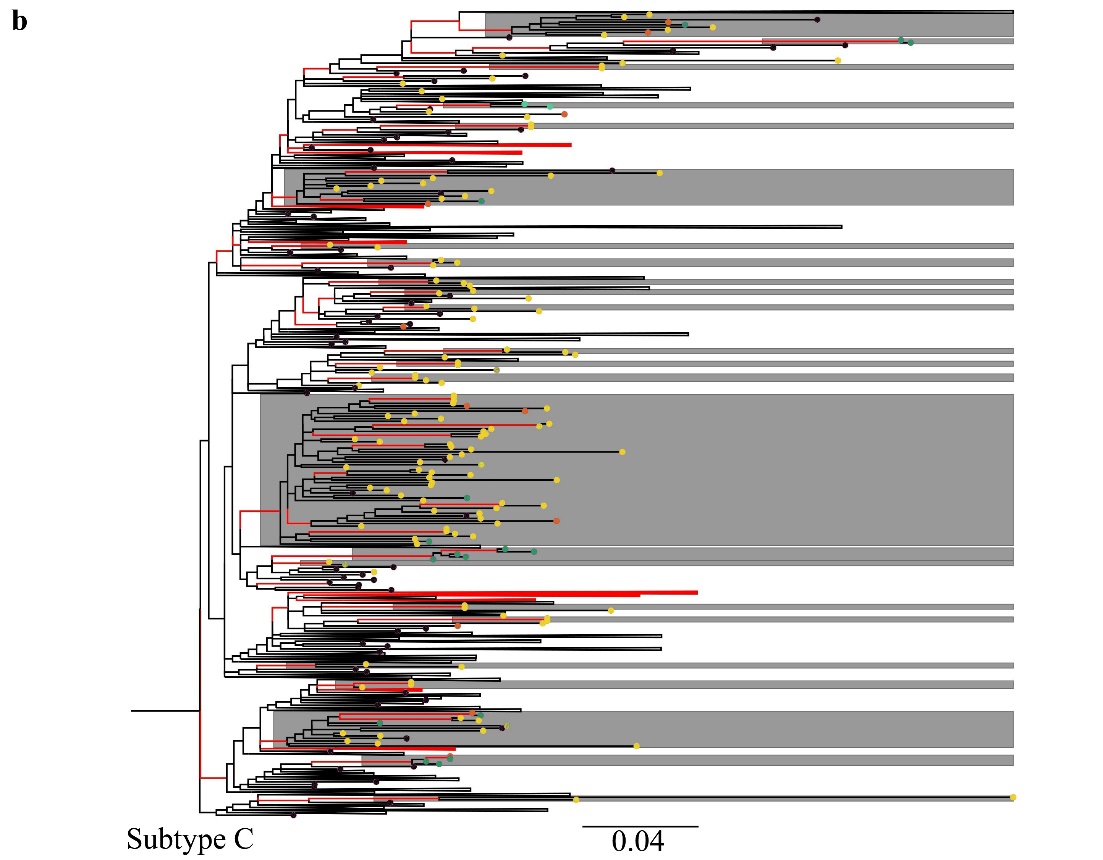
**

**
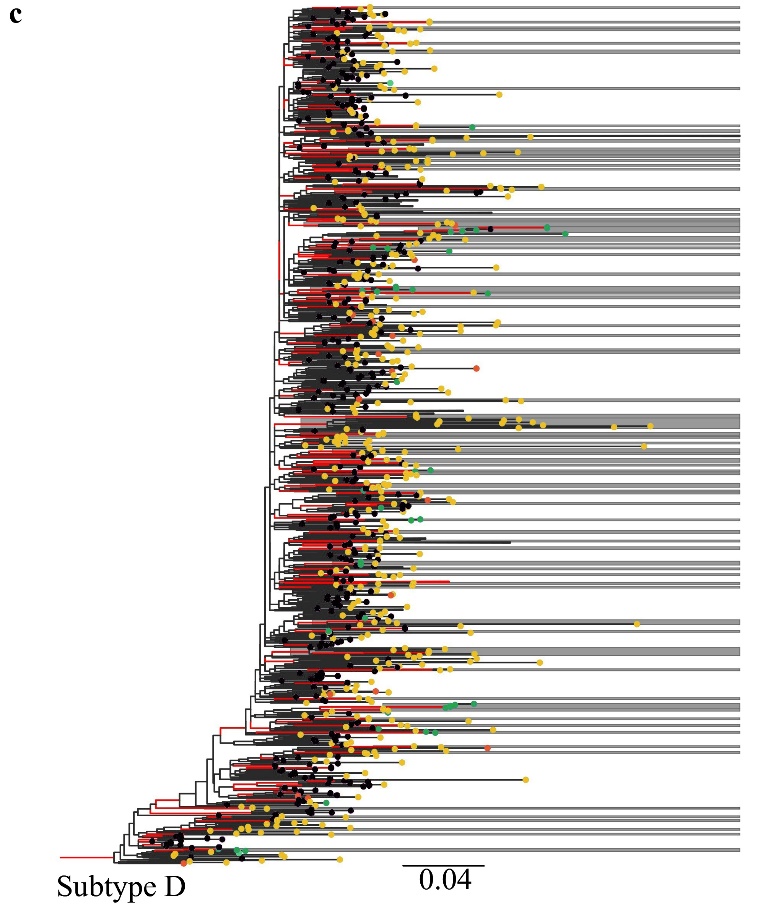
**

**Figure S4. Distribution of clusters (N=409) of different subtypes by cluster size.**

Size and subtype distribution of 409 Kenyan HIV-1 clusters identified in this study. The number of clusters per subtype are shown in the Y-axis (coloured by subtype: Red; sub-subtype A1, Brown; subtype C, and Deep Blue; subtype D clusters). The number of sequences per cluster is depicted in the X-axis.

**
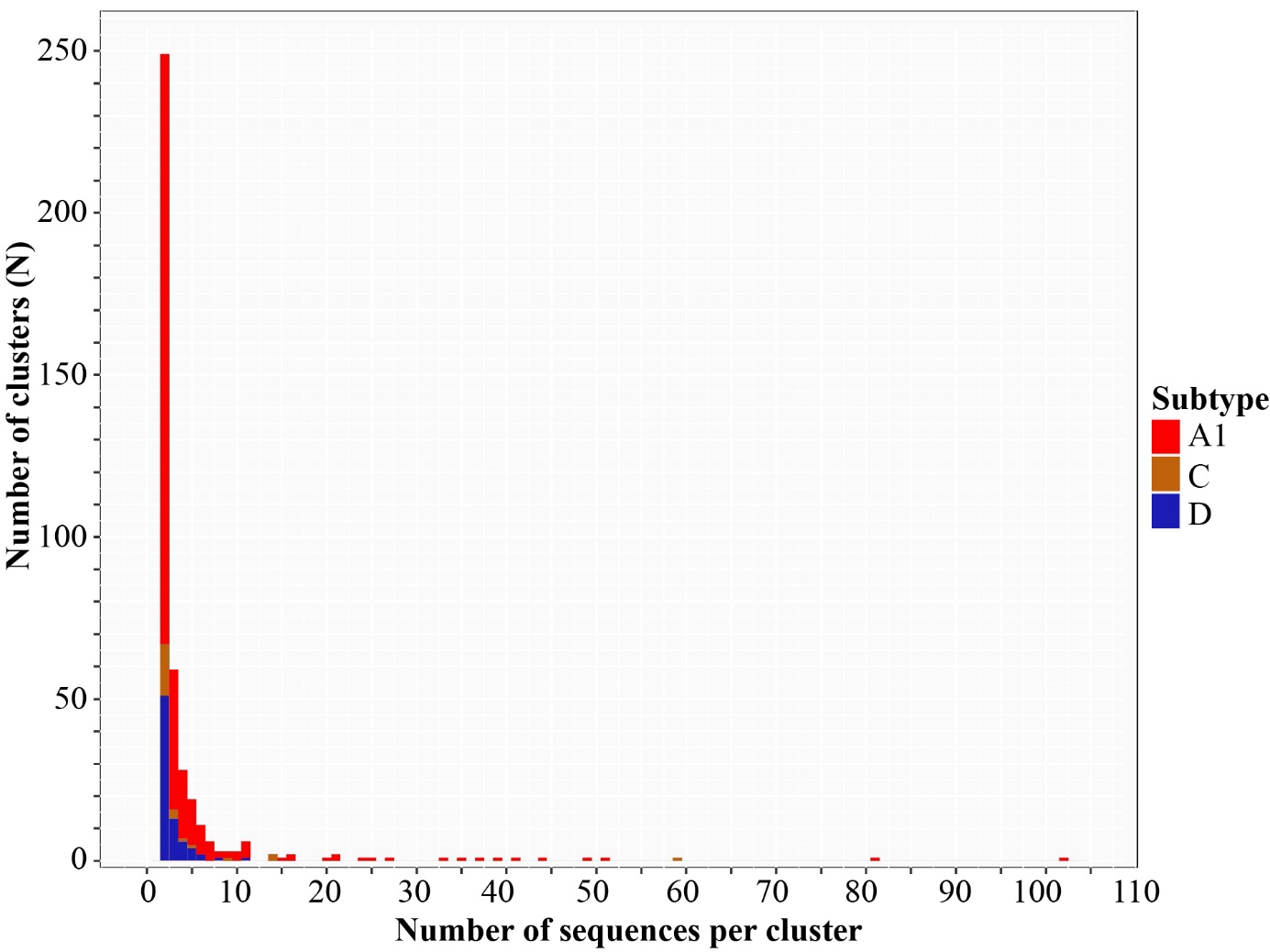
**

**Figure S5. Summary of Kenyan clusters (N=409) by geographic and risk group.**

Graphical summary of the distribution of 409 Kenyan clusters by (**a**) geographic locations (i.e. province) and (**b**) risk groups.


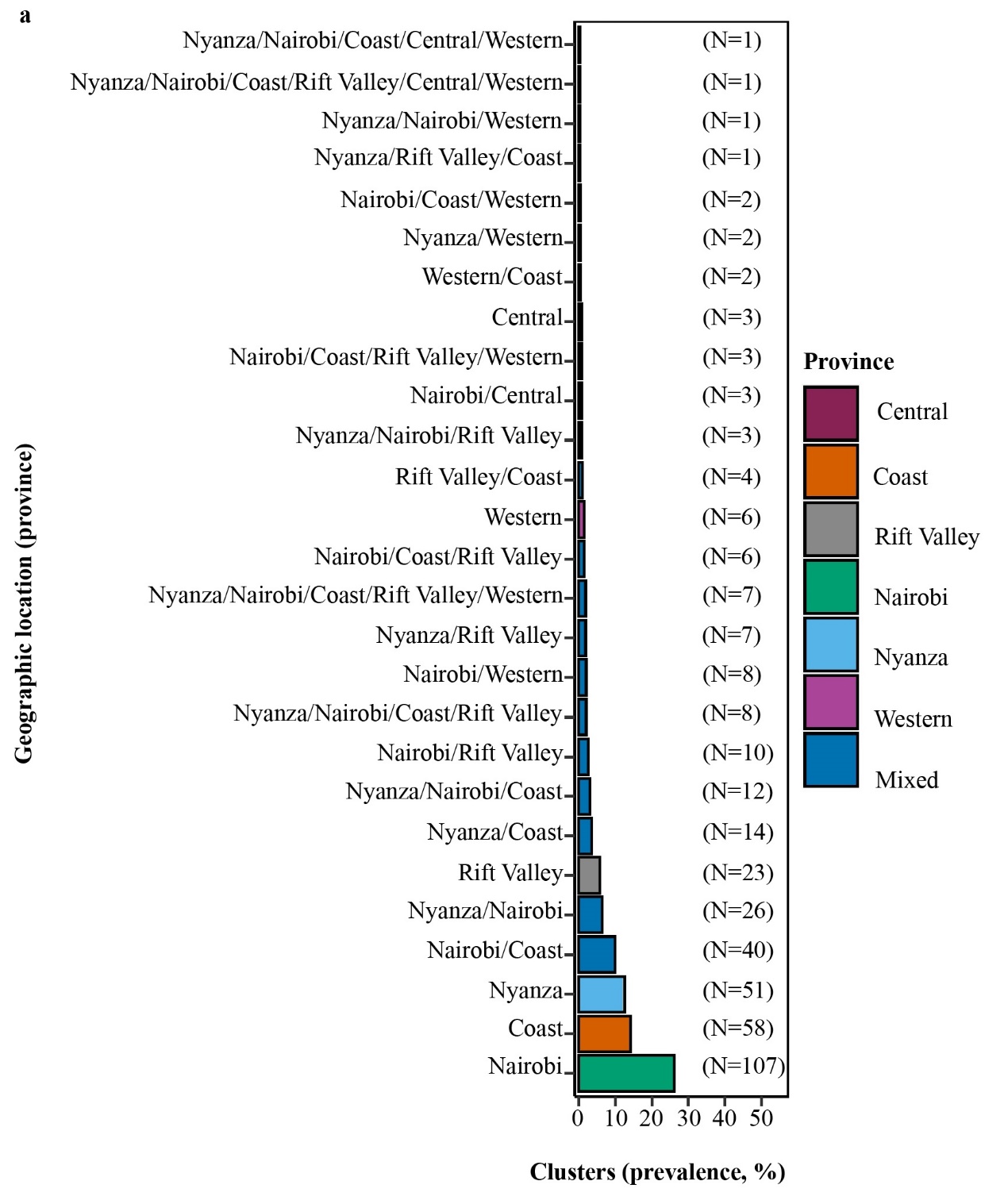


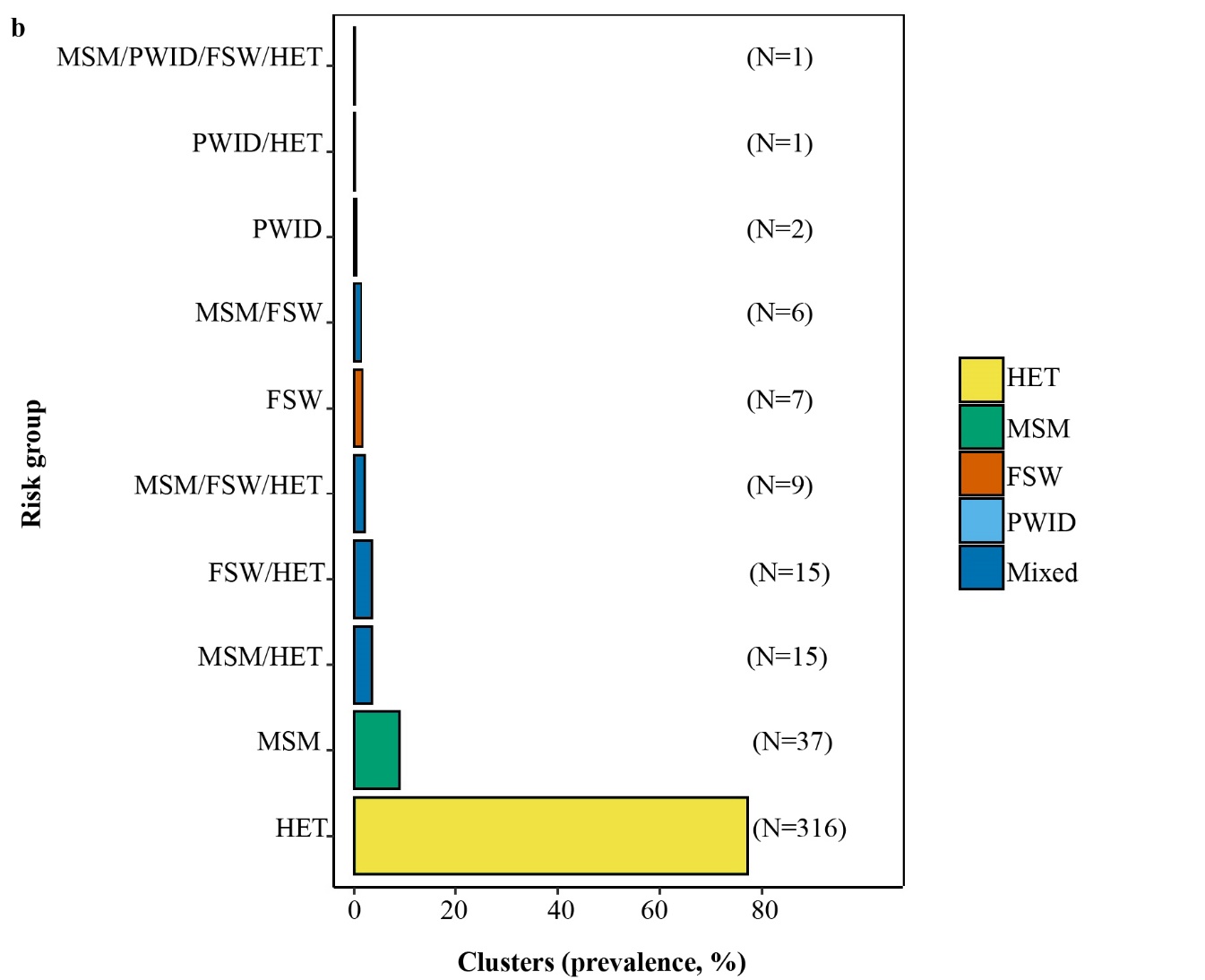


**Figure S6. Root-to-tip** **regression analyses of phylogenetic temporal signal.**

Root-to-tip regression analyses of phylogenetic temporal signal for sub-subtype A1, subtype C, and subtype D sequences from Kenya. Correlation and determination coefficient (R2) were estimated with TempEst.


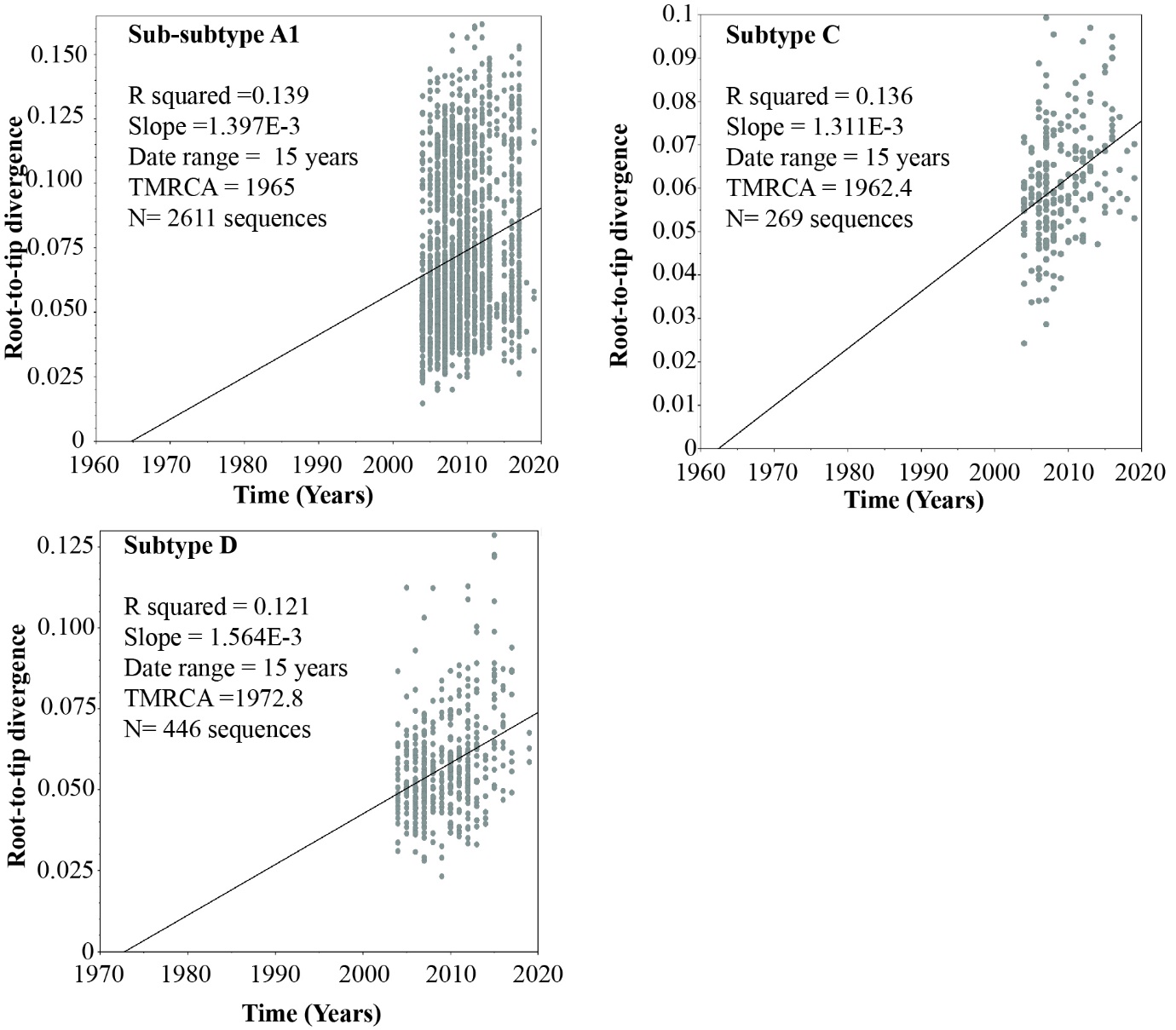


**Figure S7. Population dynamics in the HIV-1 epidemic among HET and mixed-risk group clusters.**

Bayesian Skygrid plots showing historical population dynamics of the main (**a**) HET and (**b**) mixed-risk group HIV-1 sub-subtype A1 clusters. Median estimates of the number of individuals contributing to new infections over time are shown as a continuous black line. The shaded area represents the 95% higher posterior density intervals of the inferred effective population size. Figure legends highlight information on dominating risk group per cluster, and the provinces of sampling.


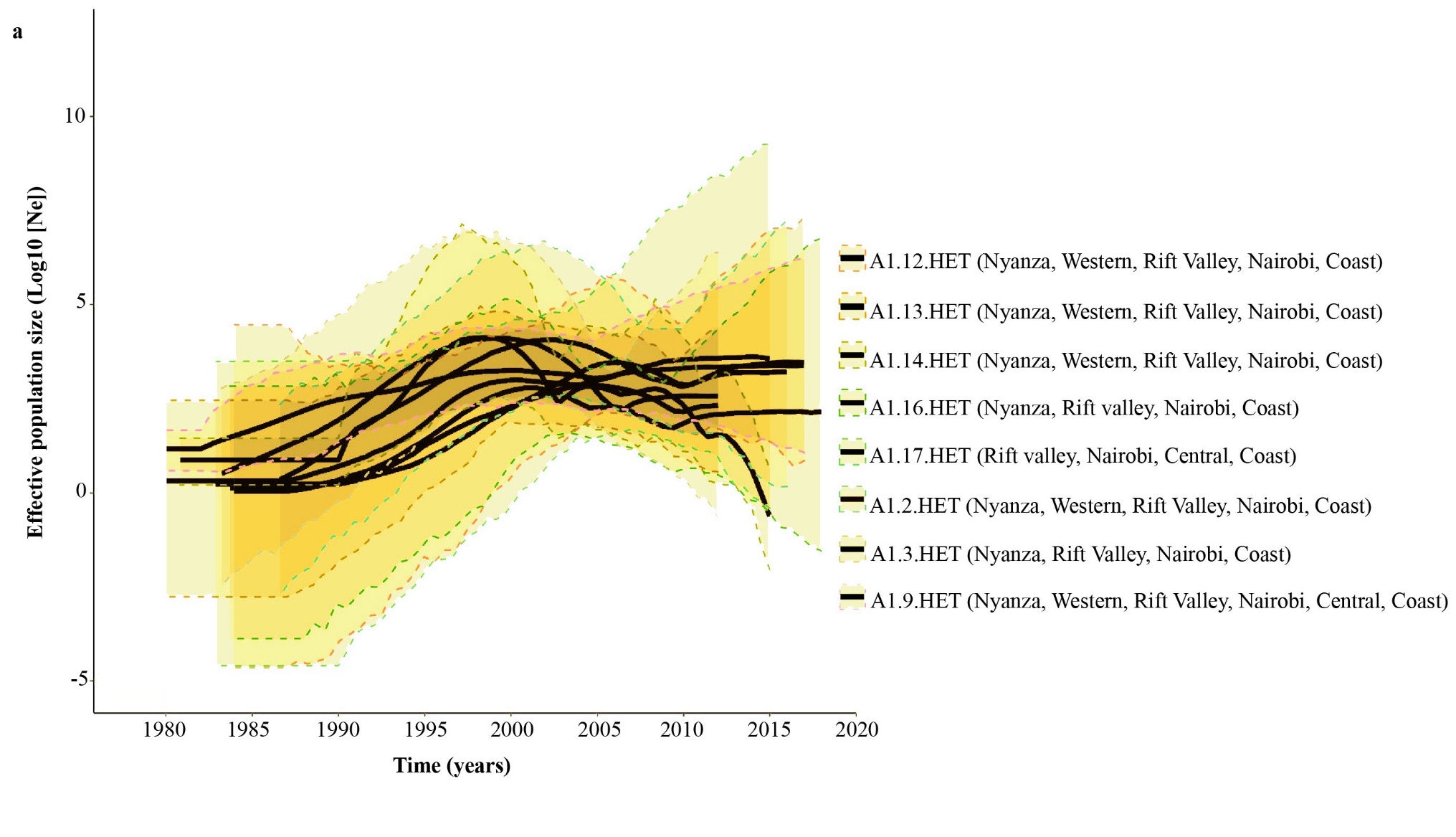


**
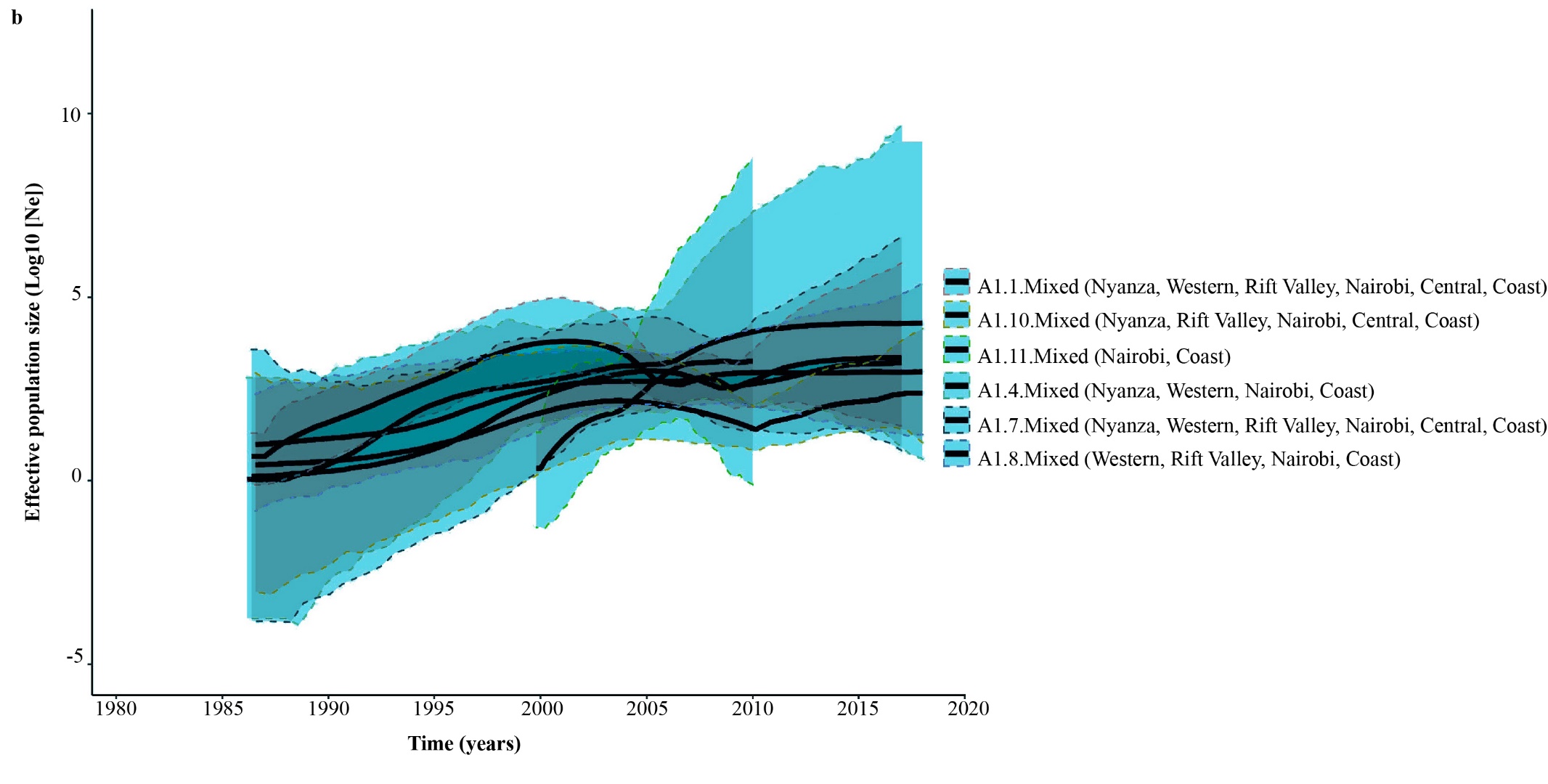
**

**Figure S8. Number and direction of HIV-1 jumps between geographic locations and risk groups based on uniform and proportional sub-sampling of 1147 HIV-1 sub-subtype A1 sequences sampled 2010-2019 in Kenya.**

Pirate plots quantifying direction and number of HIV-1 jumps between geographic locations and risk groups. Graphs represent (a) virus jumps between geographic provinces based on proportional sub-sampling (n=5 datasets; 70 sequences from Nyanza, 34 sequences from Rift Valley, 26 sequences from Nairobi, and 14 sequences from Coast); (b) virus jumps between geographic provinces based on uniform sub-sampling (n=5 datasets; 25 sequences from Nyanza, 25 sequences from Rift Valley, 25 sequences from Nairobi, and 25 sequences from Coast); (c) virus jumps between risk groups based on proportional sub-sampling (n=5 datasets; 64 sequences from HET, 14 sequences from FSW, 15 sequences from MSM, and 4 sequences from PWID); and (d) virus jumps between risk groups based on uniform sub-sampling (n=5 datasets; 27 sequences from HET, 27 sequences from FSW, 27 sequences from MSM, and 27 sequences from PWID). Black lines in the plots represent median jumps estimates (and 95% confidence intervals). The geographic plots are coloured as per the direction of transmission (blue: West-to-East; and green: East-to-West) whilst the risk group plots are coloured as per the source risk group (green: MSM; sky blue: PWID; vermillion: FSW; and yellow: HET). Overall, there was significantly more West-to-East virus flow than from East-to-West (p<0.0001; both uniform and proportional sub-sampling), and more virus flow from HET-to-key populations than vice-versa (p<0.001; both uniform and proportional sub-sampling).


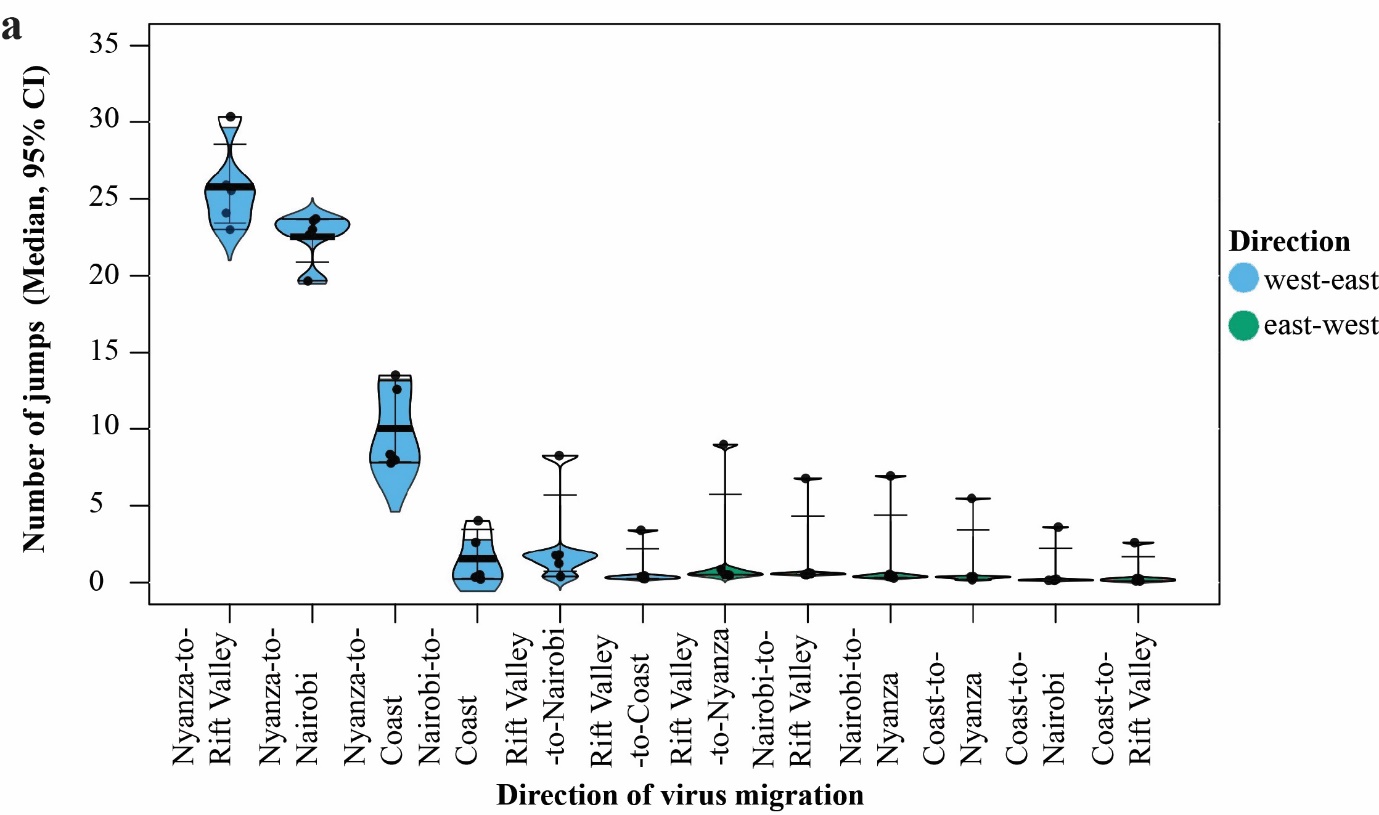


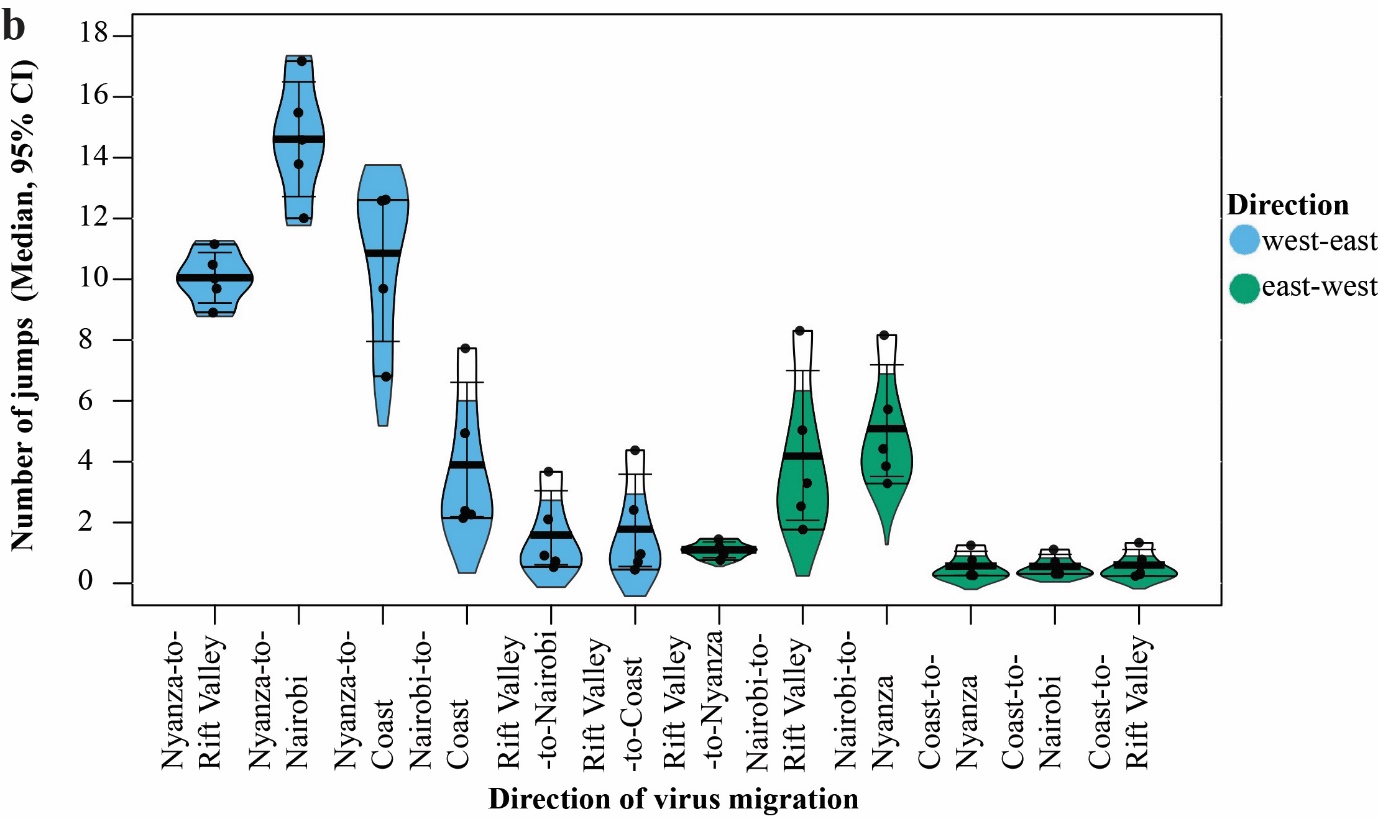

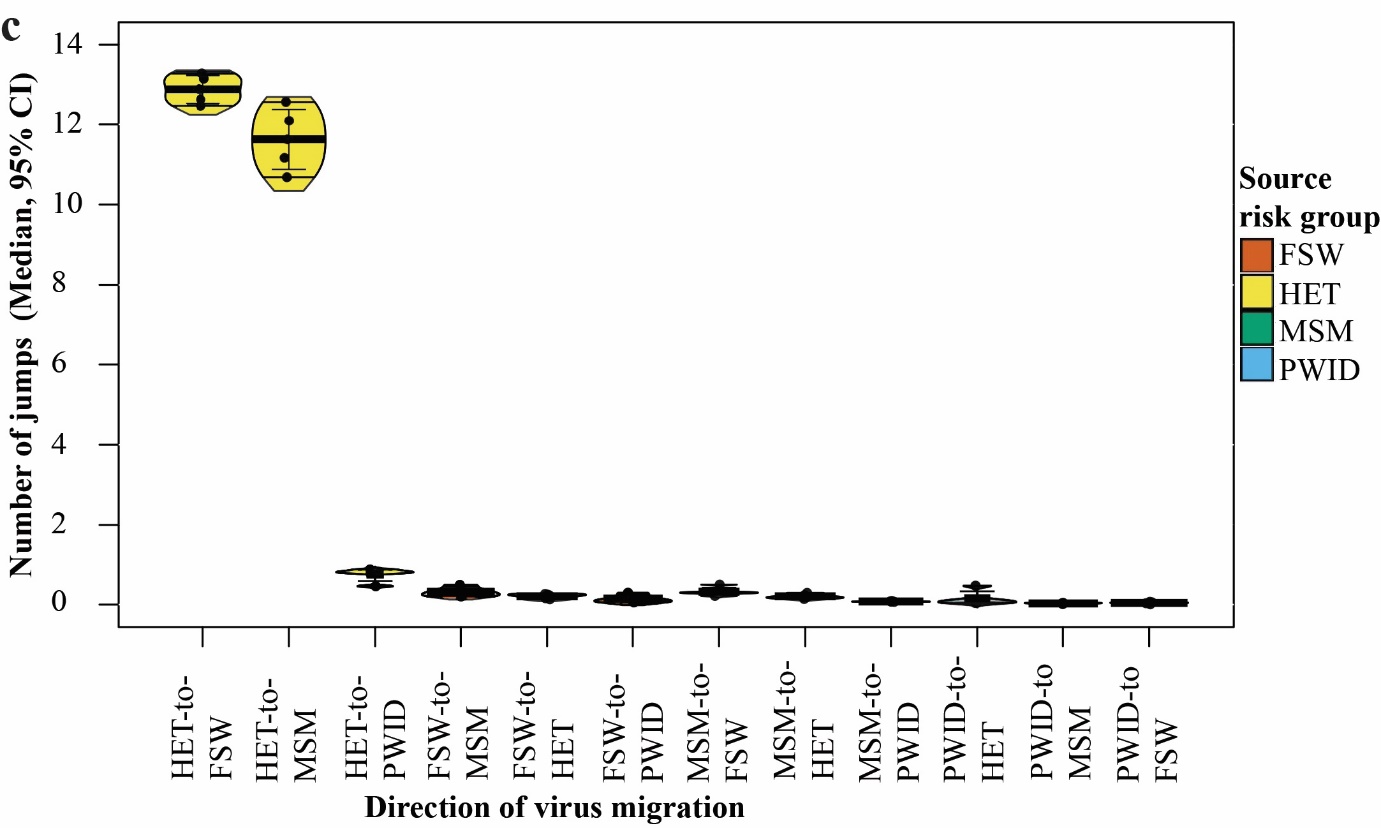

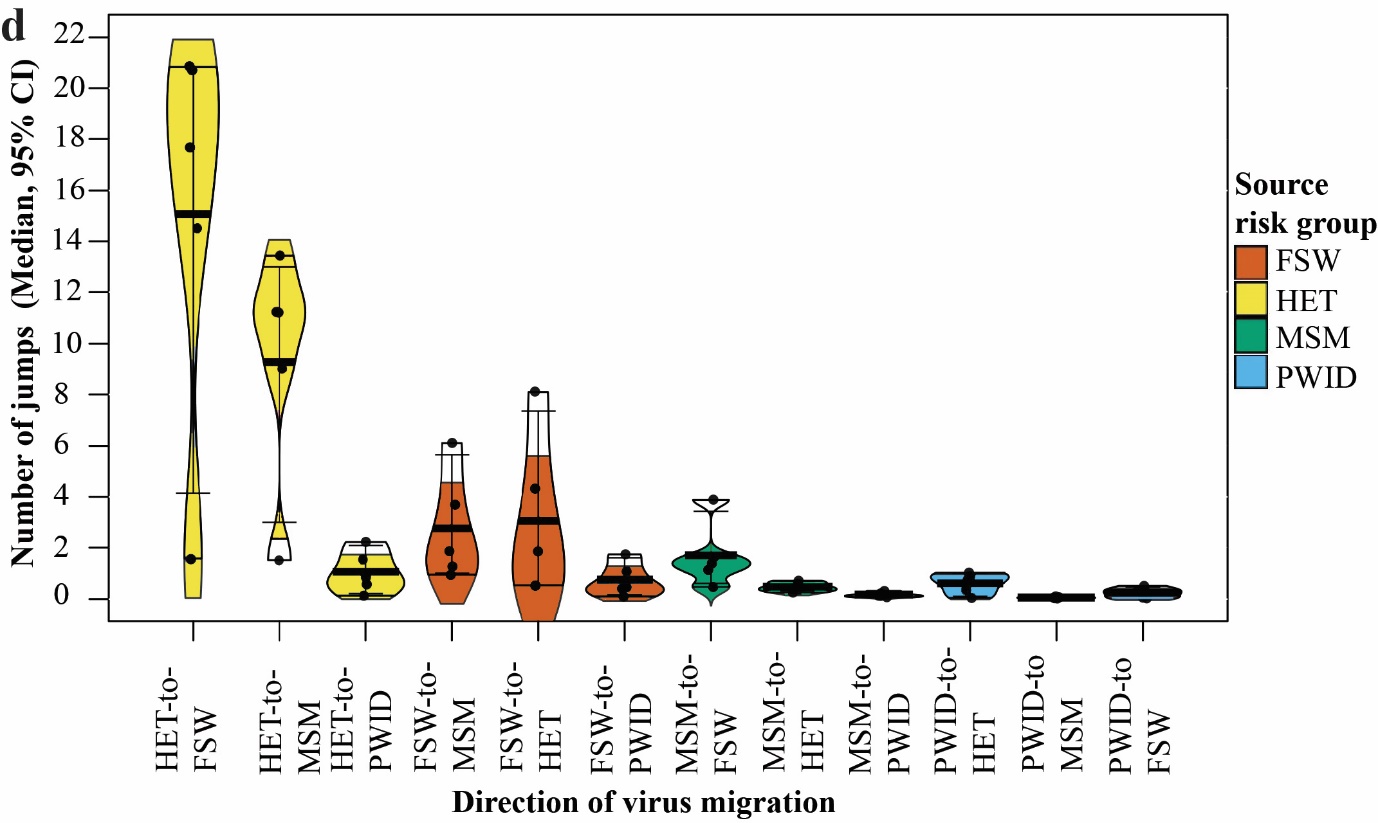


**REFERENCES**

Kenya National AIDS control council (NACC). (2018, 2019). Kenya HIV estimates report 2018. Retrieved from <https://nacc.or.ke/wp-content/uploads/2018/11/HIV-estimates-report-Kenya-20182.pdf>

Kenya National Bureau of Statistics. (2019). 2019 Kenya population and housing census Volume 1: Population by county and sub-county. Retrieved from <https://www.knbs.or.ke/?wpdmpro=2019-kenya-population-and-housing-census-volume-i-population-by-county-and-sub-county>

National AIDS and STI Control Programme (NASCOP). (2019). Key Population Mapping and Size Estimation in Selected Counties in Kenya: Phase 1. Retrieved from <https://hivpreventioncoalition.unaids.org/wp-content/uploads/2020/02/KPSE-Phase1-Final-Report.pdf>

National AIDS and STI Control Programme (NASCOP). (2020). Preliminary KENPHIA 2018 Report. Retrieved from <https://www.nascop.or.ke/kenphia-report>
